# Work orientations and turnover

**DOI:** 10.1007/s00148-026-01176-w

**Published:** 2026-06-06

**Authors:** Milena Nikolova, Juliette de Wit

**Affiliations:** 1 University of Groningen, Faculty of Economics and Business, Nettelbosje 2, 9742AE Groningen, The Netherlands; 2 Global Labor Organization (GLO), Essen, Germany; 3 The Brookings Institution, Washington, DC, USA; 4 IZA@LISER, Luxembourg, Luxembourg

**Keywords:** Work orientations, Quit intentions, Job search, Turnover, J24, J28, J81, M59

## Abstract

Voluntary turnover imposes substantial costs on organizations, yet we know little about whether workers’ fundamental orientations toward work shape their mobility decisions. In this paper, we distinguish between job, career, and calling orientations, defined as the long-term beliefs about the role of work in one’s life. Using original survey data from the Dutch LISS panel, we show that work orientations are strong predictors of quit intentions and on-the-job search, even after controlling for socio-demographic characteristics, personality traits, job characteristics, and job satisfaction. Career-oriented individuals exhibit significantly higher quit intentions and search activity, particularly when perceived promotion opportunities are limited. Calling-oriented individuals report substantially lower quit intentions and on-the-job search. By contrast, having a job orientation is not systematically associated with turnover-related outcomes. The associations between work orientations and actual job quits one and two years later are weaker, suggesting that work orientations primarily shape turnover intentions and preparatory search behavior, while actual working conditions more strongly influence mobility. Overall, our findings identify work orientations as a novel and quantitatively important determinant of turnover-related behavior and highlight the role of workers’ underlying motivations in shaping mobility beyond standard economic predictors.

**Supplementary Information:**

The online version contains supplementary material available at https://doi.org/10.1007/s00148-026-01176-w.

## Introduction

Voluntary turnover is a key issue for organizations because of the costs it imposes (Kuhn & Yu 2021; Li et al. 2022).^1^ Furthermore, turnover can be contagious, as coworkers’ job-search behavior triggers job quitting among other employees (Felps et al. 2009; Hoffmann & Vladimirov 2025). When many employees leave an organization, the remaining workers may experience reduced socialization (Gamba et al. 2024) and increased stress (Knight et al. 2013). While a certain level of turnover allows workers to move to their most productive employment (Lazear & Spletzer 2012) and enables the flow of new talent into a company, high turnover remains a key organizational issue (Glebbeek & Bax 2004).

This paper integrates insights from organizational psychology and economics to propose a new determinant of voluntary turnover: work orientations. Work orientations capture how individuals view and understand the role of work in their lives (Wrzesniewski et al. 1997). Some people view work as a job that brings a paycheck; others see it as a career that can help them achieve social status, and still others see it as a calling or a life purpose (Rosso et al. 2010; Wrzesniewski 1999; Wrzesniewski et al. 1997). *Job-oriented* individuals focus on the leisure aspects of their lives and often cannot wait to stop working, which is consistent with the traditional conceptualization of workers in economics. *Career-oriented* people view work as a means to get social recognition and status. Finally, the *calling-oriented* perceive their work as a source of fulfillment because they believe that work is personally or socially important. These work orientations, therefore, are the meanings people ascribe to work, which underpin how they carry out their work activities (Peterson et al. 2009; Wrzesniewski & Dutton 2001) and how attached they feel to their jobs. Importantly, work orientations differ from job preferences (i.e., work values), such as high pay, autonomy, or job security, which relate to desired job features (Clark and Kozák 2023; Gallie et al. 2012; Rosso et al. 2010). Instead, work orientations are the long-term dispositions about the role of work, distinct from both preferences and personality traits (openness, conscientiousness, extraversion, agreeableness, and neuroticism) (Dekas & Baker 2014).

Work orientations have primarily been studied through the lens of organizational psychology (Wrzesniewski et al. 1997). The existing evidence suggests that work orientations are likely transmitted from parents to children (Dekas & Baker 2014) and are relatively stable over short time periods (Wrzesniewski 1999). Furthermore, work orientations correlate with job satisfaction and absenteeism (Wrzesniewski et al. 1997), and the degree of similarity of work orientations in couples influences job search behavior (Jiang & Wrzesniewski 2023). This evidence suggests that how individuals view the role of work in their lives may have behavioral consequences relevant to turnover. Yet, to our knowledge, no study has investigated how work orientations influence (intended) job quits and job search.

This paper contributes to the literature by identifying a novel determinant of voluntary turnover behavior. To this end, we utilize an original module on work orientations that we collected as part of the Dutch LISS panel in 2023. The Dutch context offers a particularly informative setting to study mobility decisions (Cnossen and Lunardon 2026). As a prototypical “flexicurity” system that combines high levels of flexibility with strong worker protection (Bekker & Mailand 2018), the Dutch labor market facilitates job mobility while limiting downside risks. This is reflected in comparatively high turnover rates (Gonne 2023) and widespread flexible working-time arrangements (Goos et al. 2022). A striking example is the “small Great Resignation” that took place in 2022, when approximately one in five workers switched jobs in the Netherlands (UWV 2023).

We find that work orientations explain a substantial share of the variation in quit intentions and job search behavior, even after controlling for socio-demographic characteristics, personality traits, and job satisfaction. Yet, they matter more for intended job quits and job search than actual job quits a year later. We furthermore find that the career-oriented are likely to quit if they do not see promotion possibilities. However, we do not find evidence that the job quit behaviors of the job- and calling-oriented depend on pay satisfaction or experiences of work meaningfulness on the job. Taken together, these findings suggest that work orientations can offer an additional lens to better understand workplace behavior.

## What are work orientations?

Work orientations are individuals’ beliefs about the role of work in their lives. Specifically, they capture whether individuals view work primarily as a source of income (job orientation), a path to advancement (career orientation), or a cause of personal meaning (calling orientation) (Wrzesniewski et al. 1997). Bellah et al. (1985), who originally discussed the work orientations framework in sociology, argued that these orientations are distinct and exist within and across occupations. A key assumption is that work orientations are person-specific rather than job-specific: individuals facing the same working conditions can have different work orientations.

Early studies on work orientations treat them as relatively stable traits (Dekas & Baker 2014; Wrzesniewski 1999). However, subsequent research suggests a more nuanced view. While work orientations are stable over shorter periods (e.g., 6 months), they may evolve during life stages or career transitions (Dobrow 2013; Schabram et al. 2023; Zhang et al. 2021). Yet, it remains unclear to what extent organizations can actively shape work orientations or how personal beliefs interact with workplace environments. Accordingly, we conceptualize work orientations as relatively stable but not immutable motivational dispositions. Our work does not address their formation, stability, or change over the life course; instead, it highlights their behavioral relevance and aims to stimulate future research on the topic.

Wrzesniewski et al. (1997) explored the differences in work orientations using psychometric methods. They showed that individuals predominantly fit into one of three work orientations and that these orientations determine work satisfaction. While some studies suggest that work orientations are indeed distinct (Mantler et al. 2022; Wrzesniewski et al. 1997), others suggest that people may simultaneously have more than one work orientation (Schabram et al. 2023). Our research design is, in principle, agnostic and allows for both possibilities.

Furthermore, work orientations are theoretically distinct from several related concepts (Nikolova 2026). First, they differ from *work values*, which concern the specific outcomes people wish to achieve through work, such as high pay or autonomy (Clark 1997, 2010; Clark & Kozák 2023; Gallie et al. 2012; Kalleberg & Marsden 2013, 2019). Whereas work values capture the importance attached to specific job attributes, work orientations are about the fundamental understanding and relation to work itself.

Second, work orientations differ from *work centrality*, which refers to the importance of work relative to other life domains, such as religion, hobbies, and volunteering (Haller et al. 2023). Work centrality captures the salience of work in a person’s overall life. By contrast, work orientations describe the perceived purpose of work, i.e., whether it is seen primarily as a source of income, advancement, or meaning, i.e., regardless of how central work is compared to other domains.

Third, work orientations are distinct from *work ethic*, which reflects a moral commitment to work itself (Congleton 1991). Work ethic involves an internalized norm to work hard regardless of extrinsic rewards. Work orientations, in contrast, concern beliefs about the broader purpose work serves in one’s life. For example, a job-oriented individual may possess a strong work ethic and work diligently out of a sense of duty. In contrast, a calling-oriented individual may exert great effort because the work is intrinsically meaningful rather than out of a moral obligation (Nikolova 2026).

Finally, we distinguish between *work meaningfulness* and having a calling orientation. Work meaningfulness refers to the *experience* of work as personally or socially significant, arising from the interaction between people’s needs from working (e.g., autonomy, competence, and relatedness) and the working environment (Cnossen & Nikolova 2025; Nikolova & Cnossen 2020). In contrast, a calling orientation reflects the belief that work, as a life domain, is personally or socially important. Individuals may have a calling orientation but still view their work tasks as meaningless. This shows that, at least conceptually, having a calling orientation is a disposition, whereas work meaning is about the experience of meaning through work.

These distinctions are conceptual. To date, no dataset measures all constructs simultaneously, limiting the ability to investigate their empirical overlap.

## Determinants of job quits and job search

A substantial body of literature in economics explores the determinants of actual and intended job quits. Quit rates are typically pro-cyclical, increasing in economically advantageous times and decreasing during recessions (Akerlof et al. 1988; Hall & Lazear 1984; Lazear & Spletzer 2012). Standard economic theory emphasizes wage differentials as the primary driver, predicting that workers usually quit their jobs to get better wages elsewhere (Faberman & Justiniano 2015; Tanaka et al. 2023).

Beyond wages, a wide range of non-monetary job characteristics influences turnover (Sullivan & To 2014). These include job dis-amenities, such as occupational hazards, a lack of promotion possibilities, night shifts (Böckerman & Ilmakunnas 2009; Cottini et al. 2011), unpredictable schedules (Choper et al. 2022), fairness concerns and relative wages (D’Ambrosio et al. 2018; Dube et al. 2019; Pfeifer & Schneck 2012), a lack of employee participation opportunities (Wilson & Peel 1991), and dissatisfaction job (in)security and the nature of the work itself (Clark 2001).

A related and long-standing strand of literature uses job satisfaction as a proxy for overall working conditions to predict (intended) job quits (Böckerman & Ilmakunnas 2009; Clark, Georgellis, & Sanfey 2012; Clark 2001; Cornelißen 2009; Freeman 1978; Lévy-Garboua et al. 2007). Lower job satisfaction increases on-the-job search, which in turn predicts actual job changes, suggesting that satisfaction primarily affects turnover through search behavior (Cornelißen 2009). Workers who switch jobs typically experience short-term improvements in job satisfaction and well-being, although these improvements may fade over time (Akerlof et al. 1988; Chadi & Hetschko 2018; Nikolova 2019).

Some studies further highlight the role of organizational mission for turnover (Bode et al. 2015; Carnahan et al. 2017). For example, Portocarrero and Burbano (2024) provide experimental evidence that receiving information about the employer’s corporate social responsibility initiatives reduces quit intentions by increasing employees’ perceptions of organizational justice, i.e., the perception that their employer treats others fairly.

Recent work highlights the central role of on-the-job search and job-to-job mobility in shaping turnover. On-the-job search is widespread. In the U.S., a substantial share of employed workers actively search at any point in time, and employed job seekers receive more and better offers than the unemployed (Faberman et al. 2022). Mobility decisions are further shaped by subjective expectations, including beliefs about search costs, returns to search effort, and outside options (Miano 2025). Moreover, dissatisfaction with specific job domains predicts not only whether workers search, but also how and where they move. Delfgaauw (2007), using Dutch public sector data, shows that dissatisfaction with organization-wide aspects (e.g. management, commuting time) increases the likelihood of searching outside the employer, dissatisfaction with job-specific aspects (e.g., autonomy, career prospects) increases internal job search, and dissatisfaction with aspects with an industry component (notably job duties and work pressure) increases the likelihood of searching outside the industry. Furthermore, Cnossen and Lunardon (2026), using Dutch linked survey–administrative data, show that dissatisfaction with non-monetary job attributes is associated with both job search and job separation. They further find that dissatisfaction with wages mainly relates to job-to-job transitions, whereas dissatisfaction with work climate and work arrangements is more strongly associated with transitions into non-employment states (e.g., unemployment, sick leave, or self-employment).

Despite this extensive body of literature, existing research on job quits and search focuses primarily on economic incentives, job attributes, and overall job satisfaction, while largely ignoring individuals’ primary motivation for working. Little is known about whether and how underlying work orientations shape mobility decisions. This paper fills this gap by examining work orientations as determinants of job search and turnover, and by comparing their role to that of established job quit determinants, such as job satisfaction, working conditions, and socio-demographic characteristics.

## Conceptual framework and hypotheses

We draw on a simple conceptual framework to link work orientations and job quits (Nikolova 2026). The framework builds on standard economic models of job mobility and the preferences-for-meaning approach (Cassar & Meier 2018). As in Nikolova (2026), we adapt this framework by incorporating work orientations as a source of heterogeneity in workers’ evaluations of job attributes.

In a standard job search framework, a worker considers quitting when the expected utility from an alternative job exceeds the utility from the current job (${U}^{current}<E[{U}^{elsewhere}]$). Utility of work depends on income, W(w,e), and the cost of exerting effort on the job, C(e):

1 $$U =W\left({w}, e\right)-C(e)$$

Meaning-at-work models extend this framework by adding the utility from meaningful work (Cassar & Meier 2018; Kesternich et al. 2021; De Schouwer et al. 2025). In these models, individuals derive utility from meaningful work, M(θ,x,e). Meaning is a function of job design (i.e., mission, autonomy, competence, and relatedness), and workers differ in the importance they place on meaning. Utility, therefore, takes the form:

2 $$\begin{array}{cccc}& U=W(w,e)+M(\theta ,x,e)-C(e),& & \end{array}$$

where M represents utility from meaningful work. Here, θ reflects an individual’s preference for work meaning, while x represents work characteristics giving rise to meaning (autonomy, competence, relatedness, and mission). Workers with a high θ are willing to accept lower wages for meaningful jobs because meaning increases their overall utility, leading to lower reservation wages and higher effort. Conversely, those with low *θ* prioritize wages and show little response to meaning.

Nikolova (2026) adds a component for career prospects to the Cassar and Meier (2018) framework, reflecting the role of advancement opportunities and status concerns in workers’ utility. Thus, the model specifies utility as a function of pay W(w,e), career prospects P(p,e), work meaningfulness M(x,e), and effort costs C(e). The importance of work attributes is not equal across individuals but rather depends on individuals’ work orientations. Work orientations, i.e., job, career, and calling, in the model are introduced via the parameters λ,v, and μ*,* respectively.

In this framework, income utility W depends on wages w and effort e; career utility P depends on promotion probabilities p and effort; and meaningfulness utility M reflects the fulfillment of autonomy, competence, and relatedness (Cnossen & Nikolova 2025; Nikolova & Cnossen 2020):

3 $$U=\lambda W\left(w,e\right)+vP\left(p,e\right)+\mu M(x,e)-C(e)$$

Importantly, *λ*, *v*, and *μ* represent distinct work orientations and do not necessarily sum to one. They capture the three independent motivational dimensions rather than weights reflecting a tradeoff. A worker can therefore place high importance on earnings, career advancement, and intrinsic meaning simultaneously.

We argue that work orientations shape workers’ attachment to their current job by affecting how they evaluate job attributes. Individuals who view work as a calling may tolerate low pay or adverse working conditions if the job provides intrinsic or social meaning. Career-oriented workers may place greater weight on promotion prospects and skill accumulation and are therefore more likely to leave when advancement opportunities are limited. Job-oriented workers, in contrast, may primarily value income as a means to finance leisure and consumption and may quit when compensation falls short of their expectations. Work orientations thus influence how workers value wages, career prospects, and non-pecuniary job characteristics, and may, therefore, predict job search and quitting behavior above and beyond traditional determinants, such as job satisfaction and socio-demographic characteristics. These considerations give rise to our first hypothesis:

**H1:** Work orientations predict quit intentions, on-the-job search behavior, and actual quits.

Furthermore, because individuals differ in how they relate to work, the effect of work orientations on job search and quitting behavior should depend on job characteristics. Work orientations shape how workers evaluate key job attributes, such as pay, promotion prospects, and meaningfulness, and therefore, affect the utility they derive from their current position. Since the utility of outside options is unobserved, we model (intended) job quits and on-the-job search as a function of interactions between work orientations and job characteristics. This captures the idea that the impact of pay, career prospects, and meaningfulness on mobility decisions depends on the worker’s underlying orientation toward work.

Standard economic models treat work primarily as a disutility compensated by wages (Cassar & Meier 2018; Spencer 2015). A strong job orientation aligns with this view, as job-oriented individuals value work mainly as a source of income rather than identity or intrinsic fulfillment. For these individuals, pay constitutes the central component of job utility. When pay satisfaction is low, the utility derived from the current job declines sharply, increasing the likelihood that the expected utility of alternative employment exceeds that of the current position. Conversely, when compensation meets their expectations, job-oriented workers have a limited incentive to search or quit. We therefore expect that:

**H2:** Individuals with a strong job orientation are more likely to intend to quit, display on-the-job search behavior, and actually quit their job when they are dissatisfied with their current pay.

Moreover, career-oriented individuals derive utility primarily from advancement, status, and upward mobility. For these workers, (perceived) promotion opportunities constitute a central component of job utility. When advancement prospects are poor, the utility from the current job declines substantially, increasing the likelihood that outside options dominate. Conversely, the career-oriented may be inclined to stay in their current job if they perceive opportunities to progress. We therefore expect that having a career orientation will be more strongly positively associated with job search and quitting behavior when promotion opportunities are perceived to be poor.

**H3:** Individuals with a strong career orientation are more likely to intend to quit, display on-the-job search behavior, and actually quit their job when they perceive limited promotion or advancement opportunities.

Finally, individuals with a calling orientation derive utility primarily from experiencing their work as meaningful and aligned with their identity. For these workers, meaningfulness constitutes a central component of job utility. When the current job provides autonomy, competence, and relatedness (Nikolova & Cnossen 2020), the utility from the job is high, reducing incentives to search or quit. When meaningfulness is lacking, however, the utility from the current position declines substantially, increasing the likelihood that alternative employment offers higher expected utility. We therefore expect that:

**H4:** Individuals with a strong calling orientation are more likely to intend to quit, display on-the-job search behavior, and actually quit their job when they perceive their job lacks meaningfulness.

## Data and measurement

### Data collection

We use data from a special module of the Dutch Longitudinal Internet Studies for the Social Sciences (LISS) collected by CenterData at the University of Tilburg, the Netherlands. The LISS is a monthly internet-based nationally representative Dutch household panel with 7,500 individuals living in 5,000 households completing computer-assisted web questionnaires (Das & Knoef 2019; Scherpenzeel 2011). Researchers can add questions to the panel and link them with the existing longitudinal demographic and labor market information of the respondents.

In April and May 2023, CenterData invited 3,428 respondents to participate in the survey called “about work.” Of these, 2,525 completed the Dutch questionnaire (available in Appendices A and B in the Online Supplementary Material), yielding a response rate of 73.3%.^2^ The median response time was 5 min. In addition to the work orientations questions (Wrzesniewski et al. 1997), we elicited information on job quitting behavior and job search, as well as work meaningfulness using the Work As Meaning Inventory (WAMI) (Steger et al. 2012), which is the most widely used scale in the psychology literature.

We drop from the analysis individuals who have a non-working status as of May 2023 (16 observations), the self-employed (276 observations), those aged 61 or older as their answers are likely influenced by looming retirement (334 observations),^3^ 81 respondents who expected involuntary job loss (because their organization will close down, the respondent will be declared redundant, the respondent will retire, or the respondent’s contract will expire). We further dropped 70 respondents with missing information on the dependent variables quit intentions and job search. The final analysis sample with job search and quit intentions comprises 1,748 individuals. As we explain below, we also determined which respondents quit their jobs 1 year later. The analysis sample with job quits one year later is 1,328 due to missing employment status information across the surveys.

### Additional LISS data

One advantage of our data collection strategy is the ability to merge in additional information from the LISS panel. We merge our module with prior data on personality traits from the LISS Personality data file (study cp22n collected in May–June 2022), which provides measures of the Big-5 personality traits (variables cp22n020–cp22n069) using Goldberg’s IPIP scale (Goldberg 1992). This module contained the most recent information on personality traits before our work orientations module. We further incorporate information from the 2023 LISS Work and Schooling module Wave 16 (data file cw23o, collected in April–May 2023) with data on occupation,^4^ job satisfaction, tenure (number of years with the same employer), public/private employee status, career advancement opportunities, working hours, and pay satisfaction.

To capture realized turnover, we merged our data with the 2024 LISS Work and Schooling module Wave 17 (study cw24q), which records individuals’ employment status in July 2024, approximately 1 year after the work orientations module, allowing us to identify actual job quits. In robustness checks (Appendix Tables C2 and C3 in the Online Supplementary Material), we extend this window using the 2025 Work and Schooling module (Wave 18, study cw25r), the latest available at the time of writing, to examine whether our results persist over a 2-year horizon. Finally, we use retrospective information on job quits and job insecurity from the 2022 Work and Schooling module as additional controls for a robustness check (Table C4).

### Dependent variables

Our analysis makes use of three dependent variables. First, we measure *quit intentions* using a standard question about the likelihood of finding a job with another firm or organization in the next 12 months. Our sample excludes individuals who are likely to experience an involuntary job loss due to the workplace closing down, retirement, redundancy, or contract expiration. The original answers range from very unlikely to very likely on a 5-point scale. We recoded the quit intentions variable as 1 “Yes” if respondents indicated they were likely (6.9%) or very likely (4.1%) to quit their job in the next 12 months, and 0 “No” if they selected any other response option (19.2% neither, 27.2% unlikely, 42.6% very unlikely). This recoding allows us to measure all dependent variables on the same binary scale. In the analysis sample, 11% of respondents report quit intentions.

Second, respondents who indicated that they were neither likely nor unlikely, very likely, or likely to quit their job received a follow-up question probing into the concrete activities undertaken to look for a job, ranging from updating one’s CV to applying for job openings. Respondents reporting that they are very unlikely or unlikely to quit were not routed to this question and are coded as not actively searching. Based on this information, we created an additional binary variable, *job search*, which indicates that 20% of respondents in the sample were engaged in such activities.

Third, we link our 2023 work orientation survey module to the 2024 LISS Work and Schooling module to observe actual job changes. *Job quit* is an umbrella variable indicating two forms of labor market mobility: (1) *Employment exit:* Respondents who worked in 2023 but were no longer working in 2024, and (2) *Job switch:* Among those who remained employed in both 2023 and 2024, workers who report a job start date in 2024, or who report a 2023 start date in the 2024 module that was not reported in the 2023 Work and Schooling module (indicating a post-survey switch), are classified as switchers. We exclude workers who reported a 2023 start date in both surveys, as these individuals switched jobs before our baseline measurement.

As a robustness check (Appendix Tables C2-C3 in the Online Supplementary Material), we also examine whether work orientations measured in 2023 predict job separations in 2024–2025 using the 2024 and 2025 LISS Work and Schooling modules. We construct a 2-year transition indicator equal to one if a respondent experienced at least one employment exit or employer switch between 2023 and 2024 or 2024–2025, and zero otherwise. The measure is defined only for individuals observed in all three waves (2023–2025). This extended observation window captures separations occurring up to 2 years after our baseline survey. While our sample restrictions exclude workers reporting involuntary separation intentions at baseline, we cannot guarantee that all transitions over this longer period remained voluntary, as some respondents may have been fired or made redundant. Among the 1,096 workers with available 2023–2025 data (62.7% of the baseline sample of 1,748), 1.8% exited employment, 10.4% switched employers, and 12.0% experienced at least one job transition (0.3% both switched and exited over the 2-year window).

Quit intentions are reasonably good predictors of future turnover and job search behavior (Böckerman & Ilmakunnas 2009; Kristensen & Westergård-Nielsen 2004; Steel & Ovalle 1984; Tett & Meyer 1993). In our analysis sample, quit intentions are moderately correlated with actual job quits 1 year later (*r* = 0.21) and moderately correlated with job search behavior (*r* = 0.56). Moreover, job search is positively correlated with actual job transitions (*r* = 0.24). Because the job-search question was administered only to respondents with at least “neutral” on the job quit scale, the correlation between quit intentions and job search partly reflects the survey routing design and should be interpreted accordingly. These patterns are consistent with prior research suggesting that while intentions are informative, they do not fully explain turnover behavior (Vardaman et al. 2015). It is therefore important to consider both actual and intended job quit measures.

### Measuring work orientations

We measured work orientations using a validated survey instrument from the psychology literature (Wrzesniewski et al. 1997).^5^ The scale originally had 18 true–false items, which in subsequent research were reduced to 10 items measured on a 5-point Likert scale. In our survey, respondents answered in Dutch to what extent each of the statements in Q4–Q13 (see Table 1 and Appendices A and B in the Online Supplementary Material) applies to them, their work, or their career. All items were measured on a Likert scale, whereby 1 corresponded to “Not applicable at all,” 3 to neutral, and 5 to “Completely applicable.”

**Table 1:** Factor analysis of work orientation items: varimax-rotated component loadings

	Component 1 (calling)	Component 2 (career)	Component 3 (job)
Q4 I enjoy talking about my work with others	0.486	0.059	0.137
Q5 My work is one of the most important things in my life	0.436	0.000	− 0.044
Q6 My main reason for working is financial: to support my family and lifestyle	0.036	− 0.004	0.891
Q8 If I was financially independent, I would continue my current work even if I wasn’t getting paid for it	0.320	− 0.012	− 0.411
Q9 My work makes the world a better place	0.425	0.096	0.078
Q10 I would choose my current line of work again if we had the chance	0.472	− 0.078	− 0.011
Q11 I expect to be in a higher-level job in five years	0.112	0.594	0.010
Q12 I view my job as a stepping stone to other jobs	0.036	0.602	0.040
Q13 I expect to be doing the same work in five years (reversed)	− 0.228	0.516	− 0.094

Notes:* N* = 1,748. We standardized all items before applying polychoric principal component analysis

To construct the work orientation measures, we performed exploratory factor analysis using varimax rotation. Because of the non-continuous nature of the items, we used polychoric principal component analysis (Olsson 1979). We standardized all items to have a mean of 0 and a standard deviation of 1. We excluded item Q7 (“I am eager to retire”) because answering this item may be difficult for respondents for whom retirement is decades into the future.^6^ We used exploratory as opposed to confirmatory factor analysis because it is unclear how excluding the “I am eager to retire” item affects the construction of the job orientation, and to explore whether the job and calling orientations load on one or separate components.

**Fig. 1:**
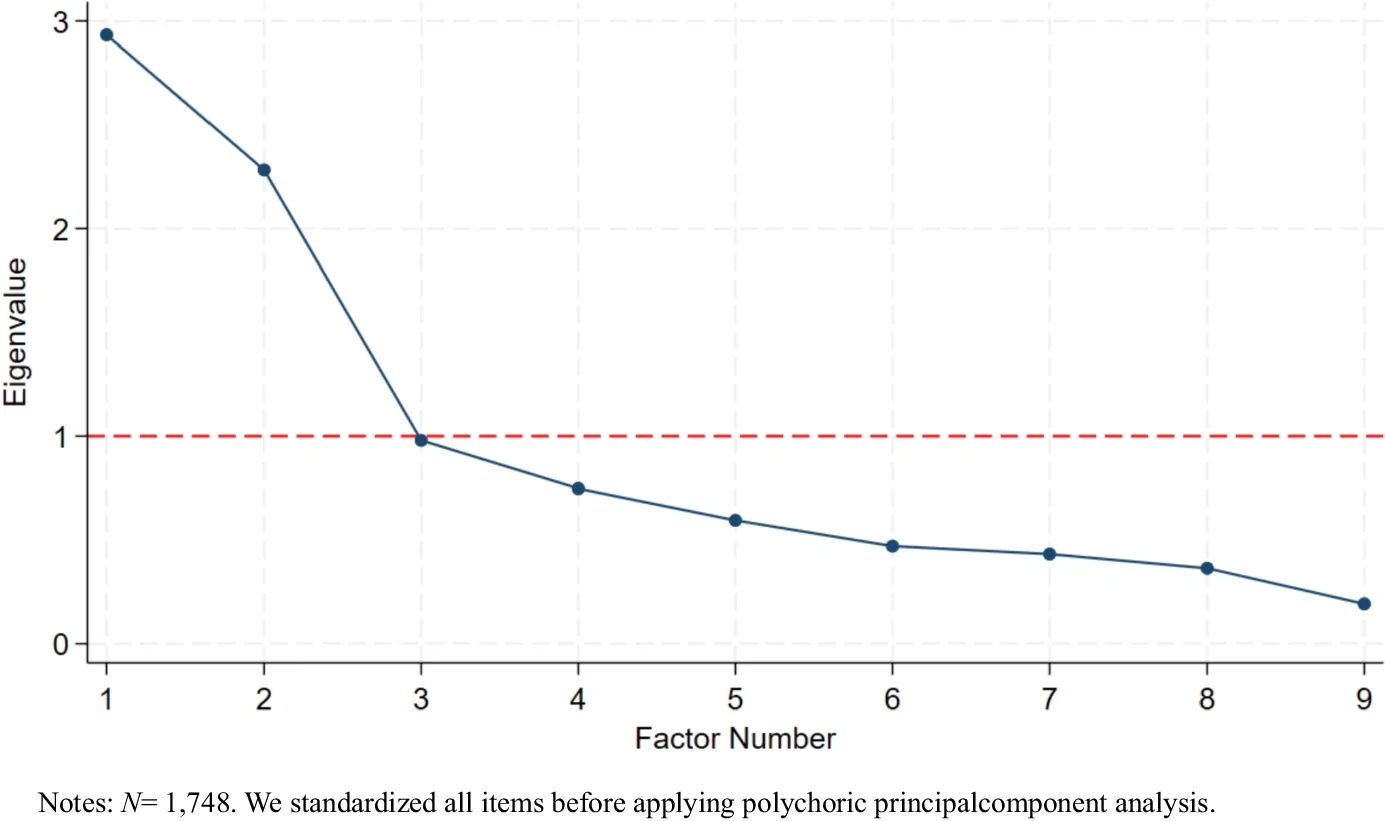
Scree plot of eigenvalues for work orientation items

We find that a three-factor solution is optimal given the scree test (Catell 1966) (Fig. 1). After varimax rotation, the eigenvalues of the three retained components are 2.74, 2.28, and 1.17, respectively, with the first component explaining 30.5% of the total variance, the second component explaining an additional 25.4%, and the third component 13% more. The three-factor solution explains 69% of the total variance in the included items, which is in line with the literature (e.g., Gandal et al. 2005).

The rotated factor loadings presented in Table 1 clearly show that items related to intrinsic meaning and purpose, such as “I enjoy talking about my work with others” and “My work makes the world a better place,” load strongly on the first component, which we interpret as the *calling orientation*. Items, such as “I expect to be in a higher-level job in five years” and “I view my job as a stepping stone to other jobs,” load on the second component, reflecting the *career orientation.* The third component captures the *job orientation*, marked by high loadings on the items emphasizing financial motives for working (i.e., “My main reason for working is financial”), and low or negative loadings on intrinsic motivation (“If I was financially independent, I would continue my current work even if I wasn’t getting paid for it”). Based on this structure, we constructed three orientation indices (calling, career, and job) using the predicted factor scores from the polychoric PCA and standardized them to have a mean of 0 and a standard deviation of 1. These are the main measures of work orientations that we use in the paper and they allow for respondents to have multiple orientations at the same time.

**Fig. 2:**
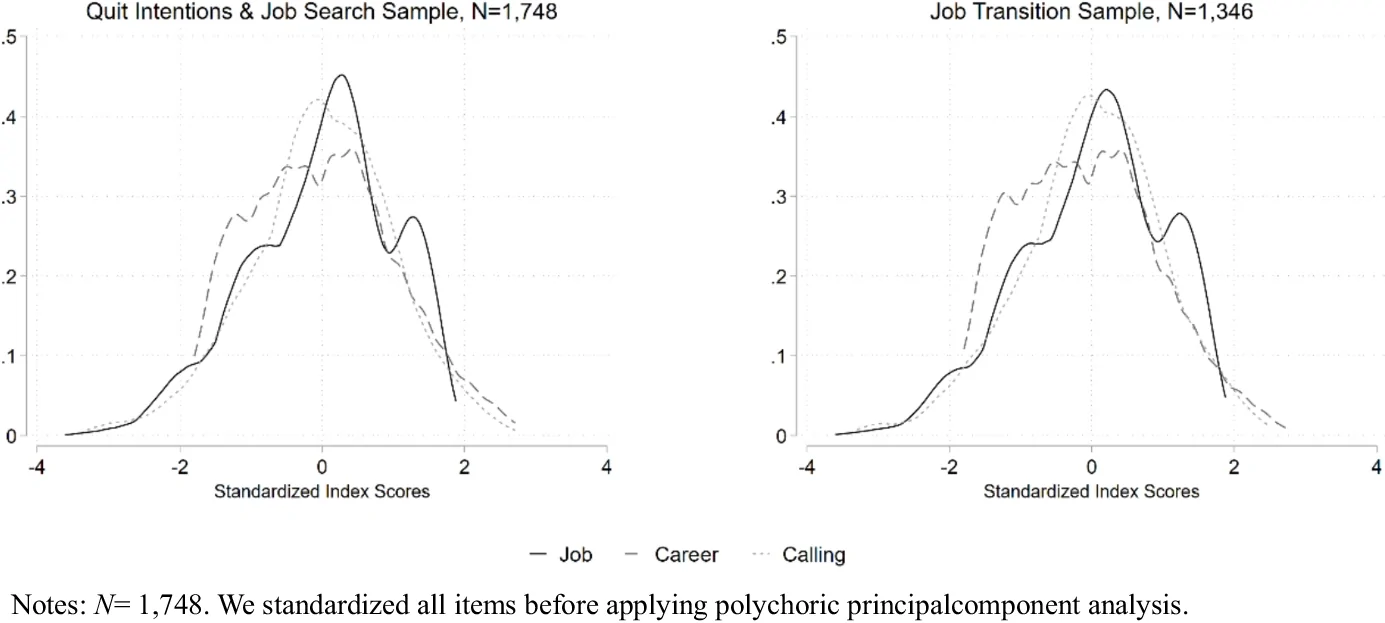
Distribution of work orientations, continuous indices

An examination of the simple correlation coefficients between the work orientation indices suggests that they are empirically distinct from one another. Specifically, the calling index is negatively correlated with the job index (*r* = –0.30) and has no meaningful correlation with the career index (*r* = 0.01). Similarly, the career and job indices are uncorrelated (*r* = 0.02). Figure 2 shows that the standardized work orientation indices are approximately normally distributed across the two analysis samples. The median scores in the main analysis sample (*N* = 1,748) are 0.126 for the job orientation, − 0.007 for the career orientation, and 0.030 for calling. The standard deviation for all three measures is 1, by construction.

**Fig. 3:**
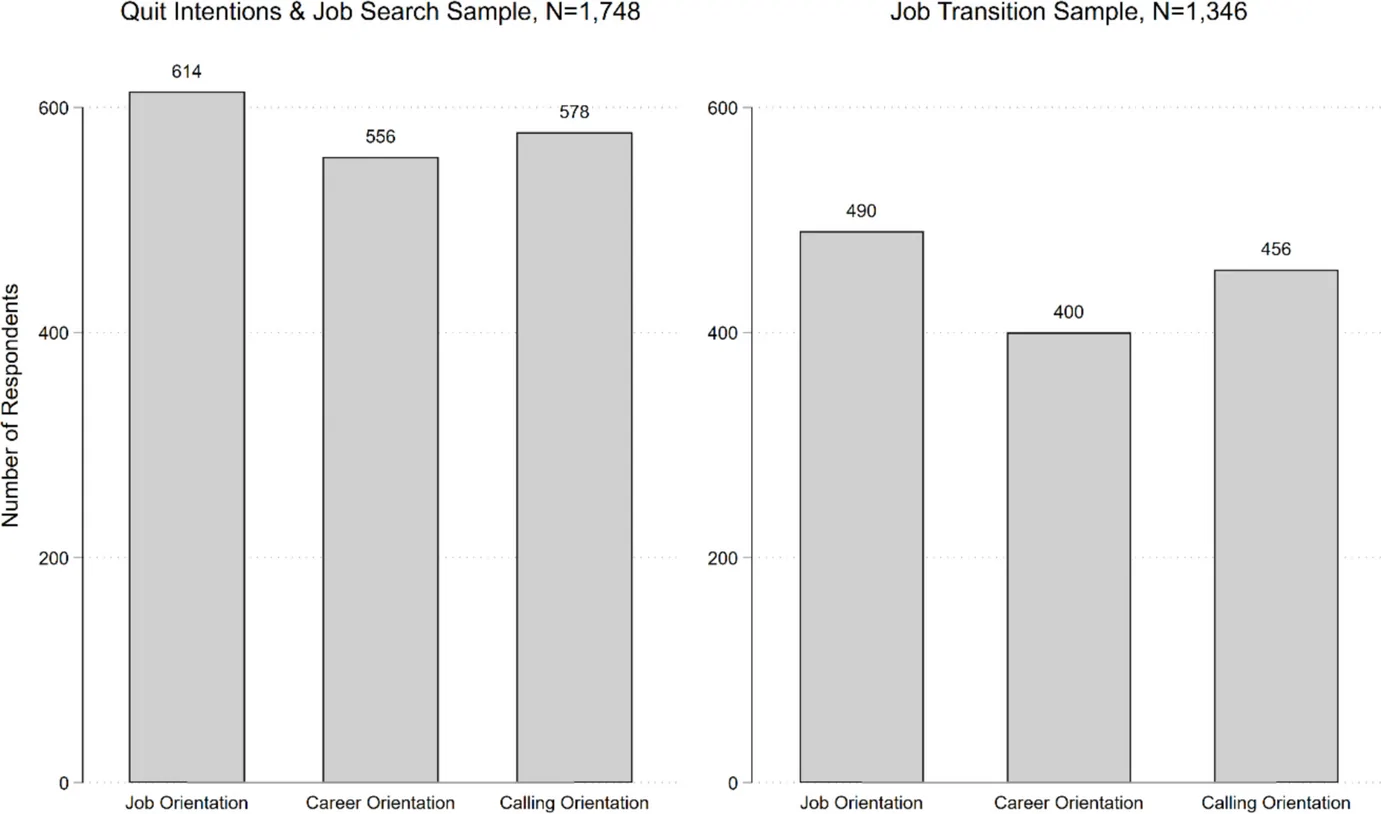
Distribution of work orientations, mutually exclusive categories

In robustness checks, we also construct mutually exclusive work orientation categories based on respondents’ highest (non-standardized) scores on the job, career, and calling indices, following Mantler et al. (2022) and Wrzesniewski et al. (1997). No respondent had identical highest scores on more than one index, so the classification does not require tie-breaking (see Fig. 3). Roughly one-third of respondents fall into each dominant work orientation category: in the full quit/search sample (*N* = 1,748), 35% are job-oriented, 32% career-oriented, and 33% calling-oriented, while in the job transition sample (*N* = 1,346), 36% are job-oriented, 30% career-oriented, and 34% calling-oriented, which is similar to the distribution in Wrzesniewski et al. (1997).

While factor analysis is standard and yields an intuitive solution, in additional analyses, we employ archetypal analysis as an alternative measurement approach (Cutler & Breiman 1994; Eugster & Leisch 2009). Archetypal analysis allows us to identify “pure types” or idealized work orientation profiles and represents each individual as a weighted combination of these archetypes, rather than assigning them to a single category or identifying latent dimensions. Using these alternative measures does not affect our main findings (see Appendix D in the Online Supplementary Material).

### Job characteristics

To test hypotheses H2–H4, we include three additional variables that capture relevant job characteristics. First, we construct a measure of *pay satisfaction* using responses to the question: “We would first like to know how satisfied you are with your wages or salary or profit earnings,” where 0 is not at all satisfied and 10 is fully satisfied. We standardize this variable to have a mean of 0 and a standard deviation of 1.

Second, we construct a measure of *perceived advancement opportunities* using the item “My prospects of career advancement/promotion in my job (are/were) poor.” The responses run from (1) “strongly disagree” (2) “somewhat disagree”, (3) “disagree” and (4) “strongly agree.” We standardize this variable to have a mean of 0 and standard deviation of 1.

Third, we measure *work meaningfulness* using the Work and Meaning Inventory (WAMI) (Steger et al. 2012), which we collected as part of the work orientations module. We conduct an exploratory factor analysis on the ten WAMI items (Q14–Q23) and retain three theoretically motivated subscales (Steger et al. 2012): (1) positive meaning (Q14–Q17), (2) meaning-making through work (Q18–Q20), and (3) greater-good motivation (Q21–Q23). For each WAMI subscale, we estimate a single-component model using principal component analysis based on polychoric correlations and compute the corresponding component scores. We then sum the three subscale scores to obtain a composite index and rescale that composite to range from 0 to 100.^7^ In the regression analyses, we standardize the final index to have a mean zero and a standard deviation of 1.

### Job satisfaction

To isolate work orientations from general job evaluations, we control for job satisfaction in many models, given its strong association with job search and turnover (Böckerman & Ilmakunnas 2009; Clark, Georgellis, & Sanfey 2012; Clark 2001; Cornelißen 2009; Freeman 1978; Lévy-Garboua et al. 2007). We use a 0–10 point Likert-scale measure of satisfaction with current work (“How satisfied are you with your current work?”) from the LISS Work and Schooling module. In robustness checks, we use an alternative 4-point Likert item (“Everything considered, I am satisfied with my job”). Both measures are standardized (mean 0, SD 1) prior to inclusion in the regression analyses.

Table C6 reports correlations between the two job satisfaction measures and individual work-orientation items. The two satisfaction measures are moderately correlated (*r* = 0.66), which indicates substantial overlap but not equivalence. Calling-related items show moderate positive correlations with job satisfaction, with the strongest association for “I would choose my current line of work again” (*r* = 0.48). Career-oriented items, such as expecting promotion or viewing the job as a stepping stone, display weak correlations with job satisfaction (*r* between − 0.15 and − 0.05). The financially motivated item is also weakly negatively correlated with job satisfaction (*r* = − 0.14). Overall, these patterns indicate that work orientations correlate only modestly with job satisfaction.

### Respondent characteristics

Table 2 reports descriptive statistics for the two analysis samples. In the main sample (*N* = 1,748), respondents are on average 43.8 years old (SD = 10.8), and 48% are male. Approximately half are married (48%) and have at least one child (52%), and 76% own their home. Just over half (53%) hold a college or university degree. The occupational distribution shows that intermediate professional roles (26%)—such as teachers, artists, and nurses—are most common, followed by clerical and support work (17%) and advanced academic or professional occupations (13%). Manual occupations (skilled, semi-skilled, and basic) together account for roughly 14% of the sample. The job transition sample (*N* = 1,346) is demographically and occupationally very similar, suggesting limited selectivity. We next examine how work orientations relate to job quits while controlling for these socio-demographic and job characteristics.

**Table 2:** Selected summary statistics, by analysis sample

	Sample:			
	Quit intentions + job search, *N* = 1,748		Job quits, *N* = 1,346	
Variable	Mean	Std. dev.	Mean	Std. dev.
Age	43.779	10.800	44.816	10.694
Male	0.479	0.500	0.494	0.500
Married	0.483	0.500	0.504	0.500
One or more children	0.515	0.500	0.523	0.500
Home owner	0.764	0.425	0.775	0.418
Higher education				
No	0.470	0.499	0.482	0.500
Yes (WO and HBO)	0.526	0.499	0.514	0.500
No information	0.005	0.068	0.004	0.061
Personal net income tertile				
Poorest	0.277	0.448	0.270	0.444
Middle	0.342	0.474	0.339	0.473
Richest	0.328	0.470	0.336	0.472
No information	0.053	0.225	0.055	0.228
Profession				
Advanced academic/professional (architect, physician, scholar)	0.134	0.341	0.139	0.346
Senior management (manager, director, company owner)	0.086	0.280	0.094	0.292
Intermediate professional (teacher, artist, nurse)	0.262	0.440	0.270	0.444
Mid-level supervisory/commercial (department manager, shopkeeper)	0.108	0.311	0.114	0.318
Clerical and support work (administrative assistant, accountant)	0.173	0.378	0.189	0.392
Skilled manual work (car mechanic, foreman)	0.050	0.218	0.056	0.231
Semi-skilled manual work (driver, factory worker)	0.055	0.228	0.063	0.243
Basic manual labor (cleaner, packer)	0.033	0.179	0.036	0.187
No information	0.100	0.299	0.038	0.191

## Empirical model

To understand the role of work orientations in influencing job quit intentions, job search behavior, and actual job quits, and to test H1, we estimate:

4 $${Q}_{i}={\beta}_{0}+{\upbeta}_{1}{W}_{i,job}+{\upbeta}_{2}{W}_{i,career}+{\beta}_{3}{W}_{i,calling}+\gamma {X}_{i}+{\epsilon}_{i}$$

where *Q*_*i*_ denotes the job quit intentions, job search, or job quitting of individual *i*. Furthermore, *W* represents work orientations, i.e., job, career, and calling, and *X*_*i*_ is a vector of control variables. We estimate specifications with progressively richer sets of controls. The baseline model includes age and biological sex. We then add marital status, children in the household, home ownership, education, income tertile, occupation, working hours, public sector employment, and tenure. Subsequent specifications further include personality traits (Big Five) and, finally, job satisfaction. Because mobility varies over the life cycle, age is included in all regressions (Burdett 1978). Because the dependent variables are binary, we estimate the baseline models using probits and report average marginal effects.^8^

To test Hypotheses 2–4, we extend the baseline specification by including job attributes, pay (${P}_{i}$), career prospects (${C}_{i}$), and work meaningfulness (${M}_{i}$), and their interactions with the corresponding work orientations:

5 $${Q}_{i}={\beta}_{0}+{\upbeta}_{1}{W}_{i,job}+{\upbeta}_{2}{W}_{i,career}+{\beta}_{3}{W}_{i,calling}+{\beta}_{4}{P}_{i}+{\beta}_{5}{C}_{i}+{\beta}_{6}{M}_{i}+{\upbeta}_{7}{W}_{i,job}\times {P}_{i}+{\beta}_{8}{W}_{i,career}\times {C}_{i}+{\beta}_{9}{W}_{i,calling}\times {M}_{i}+\gamma {X}_{i}+{\epsilon}_{i}$$

For the estimation of the interaction models in Eq. (5), we use a linear probability model to facilitate the interpretation of interaction effects, which are problematic in non-linear models (Ai & Norton 2003). We include only age and gender as controls in those models.

## Endogeneity concerns

The cross-sectional nature of the analysis raises concerns about whether it is endogeneity that entirely drives our results. Unobserved individual or workplace characteristics, such as fairness preferences or opportunities for learning new skills, may influence both work orientations and turnover behavior, and reverse causality may arise if anticipated job changes affect how individuals perceive their main motivation for working.^9^

To address these concerns, we implement several complementary strategies. Although none of these approaches can eliminate endogeneity, the consistency of the results across specifications increases confidence in the robustness of our findings.

First, we assess sensitivity to selection on observables by estimating models with progressively richer sets of controls. We begin with age and gender only, then add socio-demographic and job characteristics, followed by personality traits, and job satisfaction. The coefficient estimates on work orientations remain stable across these specifications, suggesting that the results are not driven by observable confounders.

Second, we exploit the panel structure of the data to introduce temporal separation between work orientations and actual quitting. Work orientations are measured in 2023, while actual job quits are observed 1 and 2 years later. This temporal ordering reduces concerns about reverse causality and establishes that orientations precede realized mobility outcomes. We further control for lagged quit intentions and prior expectations of job loss measured 1 year before the work orientations module, to control for pre-existing exit plans and perceived job insecurity that could simultaneously influence reported orientations and subsequent turnover.

Third, we address potential measurement concerns. Because several variables come from the same survey, common method variance (CMV) could inflate estimated associations. We mitigate this concern in three ways. First, analyses of realized job quits rely on measures observed 1 and 2 years after the work orientation measure, mechanically reducing same-instrument bias for realized turnover. Second, because CMV is often linked to stable response styles, we include personality traits to absorb systematic individual differences in reporting behavior. Finally, we estimate specifications that include job satisfaction, collected in yet another LISS module, which is a broad indicator of overall job quality and working conditions. Job satisfaction thus accounts for global job-related sentiment that could otherwise inflate correlations between orientations and turnover intentions. The persistence of the main results across specifications suggests that the findings are not solely driven by same-instrument reporting bias or generalized workplace dissatisfaction. We note that the relevance of CMV differs across hypotheses. It is most pronounced for H4, which links the calling orientation to perceived work meaningfulness, as both constructs are measured in the same survey instrument and are conceptually close. We therefore interpret the results for H4 with caution. By contrast, H2 and H3 rely on job characteristics measured in different survey modules, and analyses of actual job quits draw on outcomes observed in subsequent waves, substantially reducing CMV concerns for these hypotheses and outcomes.

Finally, our findings are robust to alternative measurement choices. Re-estimating the models using alternative samples and alternative operationalizations of work orientations yields substantively similar estimates, indicating that the findings are not sensitive to specific scaling decisions or item restrictions.

## Results

### Main results

Table 3 presents the main results concerning the association between work orientations and job quitting, in terms of average marginal effects. In panel A, we include the exogenous controls age and gender. Panel B includes additional covariates. Some of these variables (e.g., income or working hours) may be “bad controls” (Angrist & Pischke 2009) because they may be outcomes of work orientations themselves. Nevertheless, the magnitude of the coefficient estimates barely changes between panels A and B, suggesting that selection on observables and bad control problems are not the main issues behind our estimates.

**Table 3:** The relationship between work orientations and quit intentions, job search, and job quits

	Quit intention	Job search	Job quits		Quit intention	Job search	Job quits
	(1)	(2)	(3)		(4)	(5)	(6)
	Panel A: Exogenous individual controls				Panel B: All individual controls		
Job orientation	0.006	− 0.005	− 0.000		0.008	0.000	− 0.000
	(0.007)	(0.009)	(0.008)		(0.007)	(0.009)	(0.008)
Career orientation	0.098***	0.120***	0.035***		0.097***	0.120***	0.033***
	(0.008)	(0.009)	(0.008)		(0.008)	(0.010)	(0.008)
Calling orientation	− 0.060***	− 0.075***	− 0.007		− 0.065***	− 0.080***	− 0.008
	(0.007)	(0.009)	(0.008)		(0.007)	(0.009)	(0.008)
*N*	1,748	1,748	1,346		1,748	1,748	1,328
Mean DV	0.110	0.200	0.075		0.110	0.200	0.076
Pseudo *R*^2^	0.232	0.138	0.057		0.252	0.159	0.108
	Panel C: All individual controls + personality traits				Panel D: All individual controls + personality traits + job satisfaction		
Job orientation	0.005	− 0.003	− 0.001		0.006	− 0.002	− 0.004
	(0.008)	(0.010)	(0.008)		(0.008)	(0.010)	(0.008)
Career orientation	0.105***	0.118***	0.032***		0.084***	0.097***	0.022***
	(0.009)	(0.011)	(0.008)		(0.009)	(0.011)	(0.008)
Calling orientation	− 0.063***	− 0.084***	− 0.010		− 0.028***	− 0.041***	0.004
	(0.008)	(0.010)	(0.008)		(0.008)	(0.011)	(0.009)
*N*	1,526	1,526	1,228		1,454	1,454	1,217
Mean DV	0.111	0.201	0.075		0.108	0.195	0.075
Pseudo *R*^2^	0.275	0.171	0.128		0.344	0.226	0.149

Notes: The reported estimates are average marginal effects obtained after probit estimators. The individual controls in panel A are age and gender. The additional controls in panel B are marital status, children in the household, home ownership, college degree, personal income tertile, occupation, working hours, public employee status, and tenure. Panel C adds to the controls of panel B personality traits, and panel D adds job satisfaction in addition to all other controls. The work orientations indices are standardized to have a mean of 0 and a standard deviation of 1. Robust standard errors in parentheses.

*p < 0.1, **p < 0.05, ***p < 0.01

Across panels A and B, the job orientation is not statistically significantly associated with quit intentions, job search, or realized quits. By contrast, a 1 SD increase in the career orientation is associated with a 9–10-percentage-point higher probability of intending to quit (baseline: 11%), a 12-percentage-point increase in job search (baseline: 20%), and a 3-percentage-point increase in realized quits (baseline: 7.5%). The magnitudes of the associated coefficient estimates related to the regressions with intentions and search are economically large, while the effect size related to realized quits is more modest. The calling orientation exhibits the opposite pattern to that of the career one: a 1 SD increase is associated with 6–8 percentage points lower quit intentions and job search, but shows no statistically robust association with realized job quits.

We next explore whether the results are robust to including the Big-5 personality traits, which were collected approximately 1 year before our survey and are available for 1,526 out of the 1,748 respondents. The results, presented in panel C of Table 3, demonstrate that the main conclusions from panels A and B are independent of controlling for these personality traits.

Panel D includes the complete set of controls by adding job satisfaction (“How satisfied are you with your current work?”), measured on a 0–10 scale and standardized to have a mean of zero and standard deviation of 1. The sample size declines due to missing observations, yet the main coefficients remain stable. Job satisfaction is negatively associated with quit intentions, job search, and realized quits, but does not substantially attenuate the work orientation coefficients. The measurement of job satisfaction does not affect these results. If we use the 4-point Likert scale item “Everything considered, I am satisfied with my job,” the results remain unchanged (results available in Appendix Table C7).^10^ Overall, these patterns indicate that work orientations predict turnover-related behavior above and beyond both personality traits and general job evaluations.

**Fig. 4:**
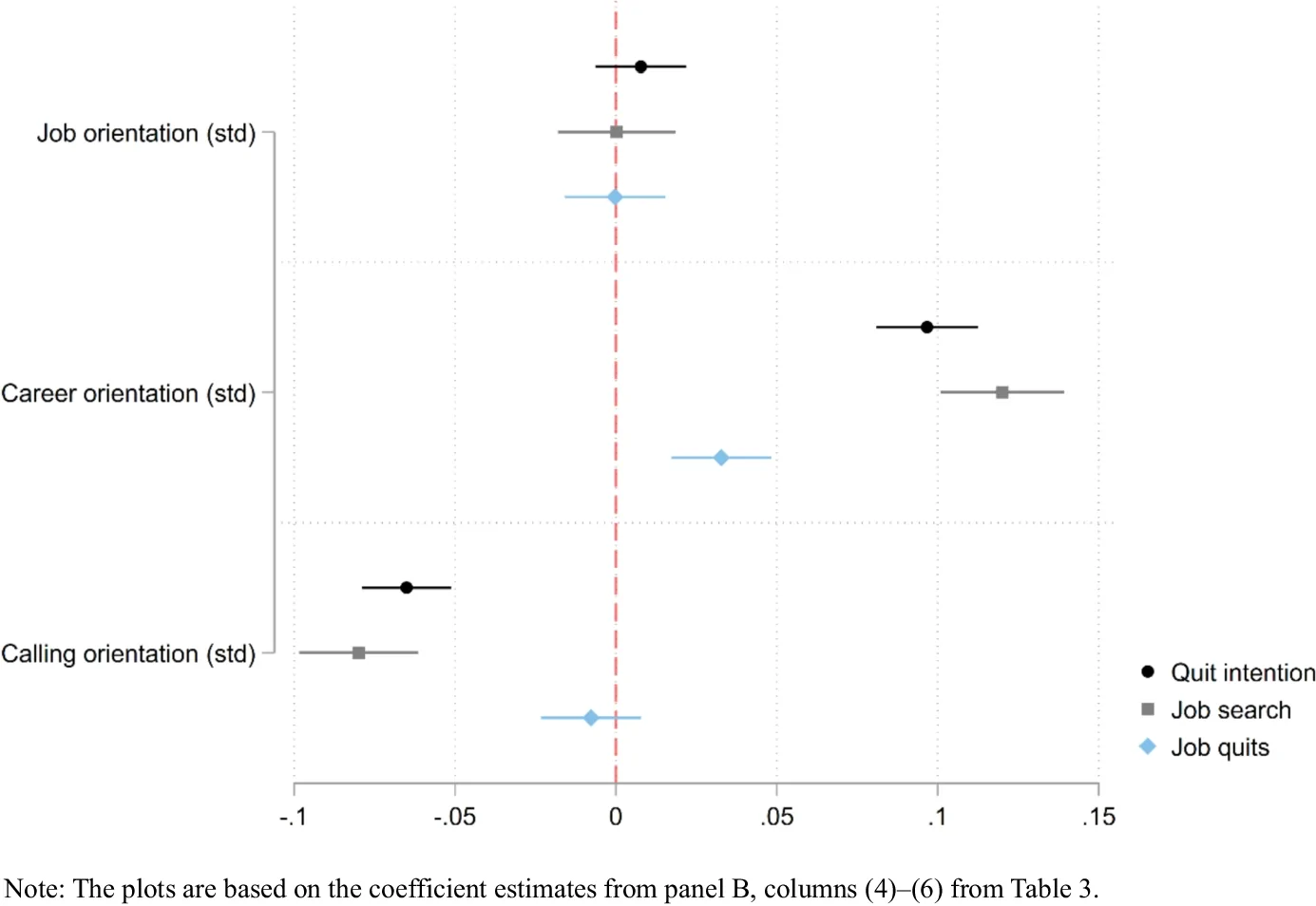
The relationship between work orientations and quit intentions, job search, and job quits

Do the estimated coefficients attenuate when moving from intentions to realized quits? Fig. 4 plots the marginal effects across the three stages of the turnover process (intentions, job search, actual quits). In absolute terms, the coefficients are largest for quit intentions and job search and smaller for realized quits. However, relative to baseline probabilities, the effects remain economically meaningful. A 1 SD increase in the career orientation raises quit intentions by roughly 88% relative to the mean of the dependent variable (0.097/0.110), job search by 60% (0.120/0.200), and realized quits by 44% (0.033/0.075). For the calling orientation, the corresponding reductions amount to approximately 59% for quit intentions, 40% for job search, and 11% for realized quits (not statistically significant).

The results thus far furnish empirical support for the conjecture that work orientations matter for job quit intentions, job search behavior, and, to a lesser degree, for actual job quits (H1). We go one step further and gauge the relative importance of work orientations vis-à-vis other factors using Shapley decompositions of the pseudo *R*^2^, which involves calculating the marginal contribution of each predictor to the pseudo *R*^2^ value across all possible combinations of predictors. This process considers the added value (in terms of percent contribution to the explained pseudo *R*^2^) of each predictor to the model’s explanatory power when it is included in combination with other factors, averaging these contributions over all possible permutations. As such, the Shapley-based decompositions determine the unique contribution of each included regressor to the overall explained variation as captured by the (pseudo) *R*^2^ (Israeli 2007; Juarez 2012; Shorrocks 2013).

**Fig. 5:**
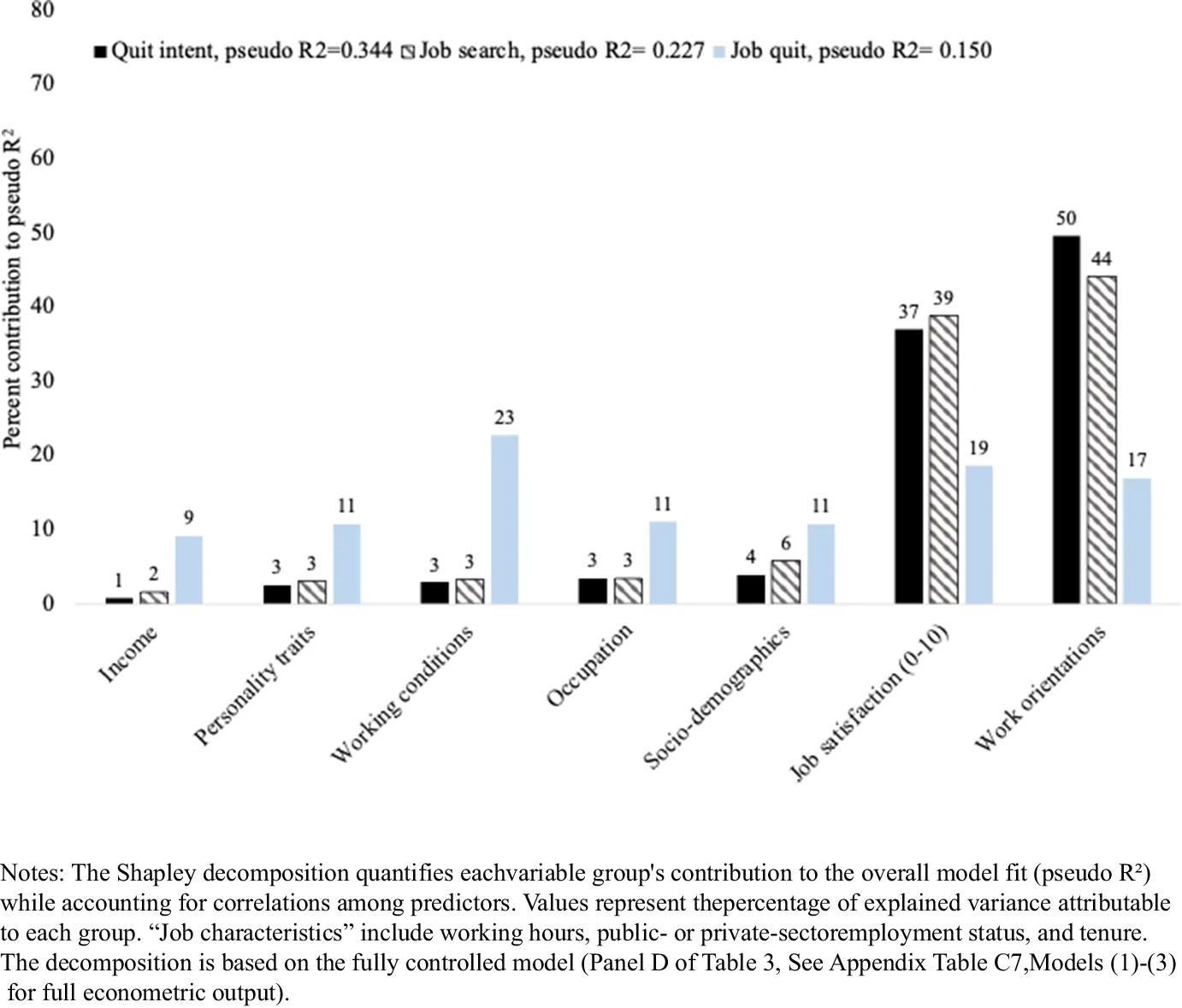
Relative contribution of variable groups to explained variance in turnover outcomes

Our Shapley decomposition results (Fig. 5) show that work orientations account for a substantial share of the explained variation in turnover intentions and search, comparable to or exceeding that of job satisfaction. For quit intentions, work orientations account for approximately 50% of the explained variation, followed by job satisfaction (37%). For job search, work orientations explain roughly 44% of the pseudo *R*^2^, while job satisfaction contributes about 39%. Thus, orientations and job satisfaction jointly dominate other predictors, with orientations contributing the largest share. When we turn to realized job quits, the relative importance shifts. Work orientations account for about 17% of the explained variation. Job characteristics, primarily tenure, public/private sector status, and working hours, contribute the largest share (approximately 23%). Although work orientations are not the dominant factor for realized turnover, they remain more important than income, personality traits, occupation, and socio-demographic characteristics.^11^

Taken together, Table 3 and Figs. 4–5 indicate that work orientations are a substantial predictor of turnover intentions and job search behavior and remain a non-trivial determinant of realized quits. The decomposition underscores that orientations matter not only statistically but also in terms of relative explanatory power.

3

### Heterogeneity

Table 4 examines heterogeneity in the association between work orientations and turnover outcomes by gender, educational attainment, and generation. These moderators capture relatively stable structural characteristics that may shape labor market opportunities and value formation.^12^

**Table 4:** Heterogeneity results by gender, educational attainment, and generations

	Job orientation			Career orientation			Calling orientation			
	Quit intention (1)	Job search (2)	Job quit (3)	Quit intention (4)	Job search (5)	Job quit (6)	Quit intention (7)		Job search (8)	Job quit (9)
*Gender*										
Male	0.011	− 0.006	− 0.003	0.102***	0.124***	0.022**	− 0.072***		− 0.079***	− 0.022*
	(0.010)	(0.013)	(0.011)	(0.011)	(0.013)	(0.011)	(0.010)		(0.013)	(0.012)
Female	0.000	− 0.003	0.003	0.094***	0.116***	0.046***	− 0.048***		− 0.070***	0.009
	(0.010)	(0.013)	(0.010)	(0.010)	(0.012)	(0.010)	(0.010)		(0.013)	(0.009)
*Education*										
No tertiary education	0.024**	− 0.001	0.003	0.099***	0.130***	0.043***	− 0.057***		− 0.079***	− 0.024**
	(0.010)	(0.013)	(0.012)	(0.011)	(0.014)	(0.013)	(0.009)		(0.012)	(0.012)
Tertiary education	− 0.008	− 0.008	0.001	0.093***	0.111***	0.029***	− 0.070***		− 0.076***	0.010
	(0.010)	(0.013)	(0.010)	(0.010)	(0.012)	(0.010)	(0.011)		(0.014)	(0.010)
*Generations*										
Gen Z/Millennials	0.014	− 0.031**	− 0.015	0.081***	0.111***	0.032***	− 0.052***		− 0.095***	− 0.011
	(0.009)	(0.014)	(0.010)	(0.012)	(0.014)	(0.010)	(0.011)		(0.015)	(0.010)
Other generations	− 0.004	0.021	0.019	0.118***	0.136***	0.041***	− 0.069***		− 0.062***	− 0.003
	(0.011)	(0.013)	(0.013)	(0.014)	(0.014)	(0.013)	(0.011)		(0.013)	(0.012)
*N*	1,748	1,748	1,346	1,748	1,748	1,346	1,748		1,748	1,346

Notes: Entries report average marginal effects from probit models. The specifications correspond to panel A of Table 3 and include age and gender as control variables. In the gender sample splits panel, gender does not feature as a control. Work orientation indices are standardized to mean zero and a standard deviation of 1. The number of observations is 1,748 for quit intention and job search and 1,346 for job quits. Robust standard errors are in parentheses

*p < 0.1, **p < 0.05, ***p < 0.01

Overall, the differences across groups are modest. The magnitude and direction of the coefficients remain broadly similar across subpopulations, suggesting that the relationship between work orientations and turnover behavior is quite stable.

For gender, the point estimates indicate that the negative association between calling orientation and quit intentions and job search is somewhat larger for men than for women. Conversely, the positive association between career orientation and realized job quits appears slightly stronger among women. However, these differences are limited in magnitude.

Educational differences are similarly moderate. The association between career orientation and job search is somewhat stronger among individuals without tertiary education, whereas the negative association between calling orientation and quit intentions appears slightly larger among college graduates. These differences suggest that meaningful work may play a somewhat stronger retention role among more educated workers, but the overall pattern remains consistent across education levels.

Across generations, the positive association between career orientation and quit intentions is somewhat smaller for Gen Z and Millennials compared to older cohorts. At the same time, the negative association between calling orientation and job search appears stronger among younger workers. Nevertheless, generational differences are limited in magnitude, and the core relationships persist across cohorts.

### Testing Hypotheses 2–4

To test H2–H4, we interact each work orientation with the corresponding job characteristic (see Eq. (5)). We estimate linear probability models using age and gender as controls. The interaction terms test whether the association between work orientations and turnover outcomes depends on pay satisfaction, perceived advancement opportunities, or work meaningfulness.

Before we discuss the results, we check whether work orientations and these job characteristics are empirically distinct. The correlations between job orientation and pay satisfaction (*r* = –0.13) and between career orientation and perceived advancement opportunities (*r* = –0.08) are small, indicating sufficient independent variation to test H2 and H3. In contrast, calling orientation and work meaningfulness are strongly correlated (*r* = 0.75). We therefore interpret these results with caution.

Table 5 reports the interaction results. The evidence provides clear support for H3: the interaction between career orientation and limited advancement opportunities is positive and statistically significant across quit intentions, job search, and realized job quits. Career-oriented individuals are therefore more likely to express turnover intentions, search behavior, and actual quitting when they perceive poor promotion prospects.

**Table 5:** Tests of Hypotheses 2–4 with interactions between work orientations and job characteristics

	Quit intention	Job search	Job quit
	(1)	(2)	(3)
Job orientation	0.005	− 0.004	− 0.005
	(0.008)	(0.010)	(0.009)
Pay satisfaction	− 0.006	− 0.022**	− 0.022**
	(0.009)	(0.011)	(0.010)
Job orientation × pay satisfaction	− 0.013*	− 0.002	0.003
	(0.007)	(0.009)	(0.010)
Career orientation	0.103***	0.119***	0.032***
	(0.009)	(0.010)	(0.009)
Poor advancement	0.050***	0.053***	0.007
	(0.008)	(0.010)	(0.008)
Career orientation × poor advancement	0.052***	0.045***	0.018**
	(0.006)	(0.008)	(0.007)
Calling orientation	− 0.041***	− 0.052***	0.008
	(0.012)	(0.015)	(0.011)
Work meaningfulness	− 0.012	− 0.007	− 0.008
	(0.011)	(0.014)	(0.010)
Calling orientation × work meaningfulness	0.012**	0.010	0.014**
	(0.006)	(0.007)	(0.007)
Age	− 0.000	− 0.000	− 0.002**
	(0.001)	(0.001)	(0.001)
Male	− 0.028**	− 0.031*	− 0.023
	(0.014)	(0.018)	(0.014)
Constant	0.122***	0.204***	0.154***
	(0.036)	(0.047)	(0.037)
*N*	1,537	1,537	1,266
*R*^2^	0.227	0.170	0.047

Notes: Estimates are obtained from OLS regressions including age and gender as controls. Pay satisfaction, perceived advancement opportunities, work meaningfulness, and all three work-orientation indices (job, career, and calling) are standardized to have a mean of 0 and a standard deviation of 1. Interaction terms use the standardized versions of both components. Robust standard errors in parentheses.

*p < 0.1, **p < 0.05, ***p < 0.01

Support for H2 is limited. The interaction between job orientation and pay satisfaction is marginally statistically significant only for quit intentions and not for job search or realized quits. For H4, the interaction between calling orientation and work meaningfulness is statistically significant for quit intentions and realized quits, but not for job search. However, the interaction coefficient is positive, implying that the negative association between meaningfulness and turnover outcomes becomes weaker (rather than stronger) as calling orientation increases. This pattern is opposite to the directional prediction of H4. Given the high empirical overlap between the calling orientation and work meaningfulness (*r* = 0.75), the interaction should be interpreted with caution. Overall, the results do not provide support for H4.

Our results show clear support for H3 but not H2 or H4. The strong and consistent interaction between having a strong career orientation and limited promotion prospects suggests that when upward mobility is constrained, the outside option becomes immediately relevant. By contrast, the mechanisms underlying H2 and H4 may be weaker for structural reasons.

First, for job-oriented individuals, pay dissatisfaction does not necessarily trigger mobility if outside wage gains are uncertain or switching costs are high. In coordinated labor markets such as the Netherlands, wage dispersion is relatively compressed (Schneck 2021), which may limit the expected payoff from mobility. Job-oriented workers may therefore tolerate moderate pay dissatisfaction as long as income remains stable. This is a hypothesis that future research should investigate.

Second, individuals with a strong calling orientation can experience low task-level meaningfulness yet remain attached due to their professional identity, professional norms, or value other job attributes. The correlation between the WAMI scale and the calling orientation is high at 0.75, suggesting that the two measures are empirically too closely aligned to generate meaningful interaction variation.

In principle, job characteristics may also moderate the behavior of workers whose dominant orientation differs from the pairing considered in H2–H4. For example, a calling-oriented individual may also value high pay. Estimating a fully saturated specification with all cross-interactions, however, would require estimating a large number of parameters and substantially reduce effective cell sizes. Given our sample size, the data do not provide sufficient statistical power for such a specification. When estimated, the fully interacted models yield no consistent additional insights. Alternatively, we estimated an additional specification using mutually exclusive orientation categories (i.e., respondents are assigned to only one category based on the highest score on the indices). This reduces dimensionality but allows us to interact each job characteristic with each work orientation group. These results, which are available upon request, provide little additional insight beyond the results in Table 5. Taken together, the evidence indicates that advancement opportunities condition turnover behavior primarily for career-oriented individuals, whereas broader cross-orientation interaction effects receive limited empirical support.

### Robustness checks

#### Non-overlapping work orientations

We classify respondents into mutually exclusive “job,” “career,” and “calling” groups based on their highest score on the work orientation indices. Table 6 reports the corresponding regression results as average marginal effects from probit models, with job orientation as the reference category.

**Table 6:** The relationship between work orientations categories and quit intentions, job search, and job quits

	Quit intention	Job search	Job quit	Quit intention	Job search	Job quit
	(1)	(2)	(3)	(4)	(5)	(6)
	Panel A: Exogenous individual controls			Panel B: All individual controls		
*Ref. group: Job orientation*						
Career orientation	0.096***	0.123***	0.026	0.087***	0.110***	0.015
	(0.022)	(0.027)	(0.019)	(0.023)	(0.027)	(0.019)
Calling orientation	− 0.060***	− 0.087***	− 0.014	− 0.065***	− 0.097***	− 0.021
	(0.015)	(0.020)	(0.016)	(0.015)	(0.020)	(0.017)
*N*	1,748	1,748	1,346	1,748	1,748	1,328
Mean DV	0.110	0.200	0.075	0.110	0.200	0.076
Pseudo *R*^2^	0.079	0.059	0.035	0.100	0.077	0.090
	Panel C: All individual controls + personality traits			Panel D: All individual controls + personality + job satisfaction		
*Ref. group: Job orientation*						
Career orientation	0.100***	0.120***	0.028	0.060***	0.093***	0.020
	(0.024)	(0.030)	(0.019)	(0.020)	(0.027)	(0.018)
Calling orientation	− 0.058***	− 0.105***	− 0.016	− 0.016	− 0.051**	0.001
	(0.016)	(0.022)	(0.017)	(0.017)	(0.022)	(0.018)
*N*	1,526	1,526	1,228	1,454	1,454	1,217
Mean DV	0.111	0.201	0.075	0.108	0.195	0.075
Pseudo *R*^2^	0.113	0.097	0.112	0.249	0.186	0.141

Notes: The reported estimates are average marginal effects obtained after probit estimators. The individual controls in panel A are age and gender. The additional controls in panel B are marital status, children in the household, home ownership, college degree, personal income tertile, occupation, working hours, public employee status, and tenure. Panel C adds to the controls of panel B personality traits, and panel D adds job satisfaction in addition to all other controls. The reference category for work orientation is “job.” *N* = 1,748. Robust standard errors in parentheses.

*p < 0.1, **p < 0.05, ***p < 0.01

The results are broadly in line with those obtained with the continuous indices in Table 3. Relative to job-oriented respondents, career-oriented individuals are significantly more likely to report quit intentions and engage in job search across specifications. Calling-oriented individuals, in contrast, are significantly less likely to report quit intentions and job search compared to the job-oriented reference group. For realized job quits, the pattern is weaker and the work orientations appear unrelated to quitting behavior, even though the patterns of coefficients remain the same. Overall, the categorical specification confirms that career orientation is associated with higher turnover intentions and search behavior, whereas calling orientation is associated with lower turnover intentions, while differences in realized quitting are not statistically significant.

#### Including the “eager to retire” item

 In the main analyses, we exclude the item “I am eager to retire” item from the Wrzesniewski et al. (1997) battery because young workers may find it difficult to provide an answer. Including this item does not change the factor solution (see Appendix C, Fig.C1) and the patterns of factor loadings (Table C8). The main results and conclusions from Table 3 also remain in this analysis (Table C9).

#### Work orientations archetypes

 We also adopt a statistical method called archetypal analysis to extract profile-like types from our dataset (Cutler & Breiman 1994; Eugster & Leisch 2009). Unlike other data reduction techniques, such as factor analysis or cluster analysis, archetype analysis focuses on the observations rather than the distributional characteristics of the variables. The archetypes can be considered as “pure types” that describe all other elements of the dataset. By construction, archetype analysis configures all individuals in a dataset as a linear combination of the archetypes. In the context of our study, the archetypes yield a configurational view of work orientations, with each archetype representing an idealized work orientation profile (inspired by Schabram et al. 2023).^13^ Details on the construction of the archetypes, as well as additional results, are available in Appendix D in the Online Supplementary .

Based on the nine work orientation items, we identify three distinct archetypes. Each archetype reflects a specific pattern in how respondents combine the items, allowing us to classify their dominant work orientation. *Archetype 1* indicates that their main reason for working is financial, to support their family and lifestyle (Q6). This is in line with the *job orientation. Archetype 2* expects to be in a higher-level job in 5 years and views their job as a stepping stone to other jobs. This aligns with the *career orientation*. *Archetype 3* indicates, among other things, that their work is one of the most important things in their lives and that they would continue in their jobs even if they were financially independent. This answer pattern aligns closely with the *calling orientation*.

We assign individuals a score (0–1) that measures the degree to which they resemble each archetype. By construction, the archetypal scores sum to 1.^14^ Using these scores, we re-estimate our main models using the archetypal analysis work orientations measures and present the results in Table 7. This table shows the average marginal effects after probit estimations. AT1, reflecting the job orientation, is the base category; using all three archetypes in the model implies multicollinearity issues. The findings are in line with our main findings in Table 3 and the robustness tests in Table 6. The career-oriented archetype is more likely to have quit intentions and search for jobs compared to the job-oriented archetype. We observe the opposite pattern for the calling archetype, which has lower quit intentions and displays less job search behaviour in comparison to the job-oriented archetype.

**Table 7:** The relationship between archetypes of work orientations and quit intentions, job search, and job quits

	Quit intention	Job search	Job quit	Quit intention	Job search	Job quit
	(1)	(2)	(3)	(4)	(5)	(6)
	Panel A: Exogenous individual controls			Panel B: All individual controls		
*Ref. group: AT1 Job orientation*						
AT2 Career orientation	0.326***	0.422***	0.121***	0.319***	0.417***	0.109***
	(0.034)	(0.043)	(0.037)	(0.035)	(0.045)	(0.037)
AT3 Calling orientation	− 0.244***	− 0.263***	− 0.021	− 0.270***	− 0.295***	− 0.028
	(0.039)	(0.049)	(0.040)	(0.040)	(0.050)	(0.040)
*N*	1,748	1,748	1,346	1,748	1,748	1,328
Pseudo *R*^2^	0.214	0.127	0.0501	0.236	0.148	0.102
	Panel C: All individual controls + personality traits			Panel D: All individual controls + personality + job satisfaction		
*Ref. group: AT1 Job orientation*						
AT2 Career orientation	0.349***	0.400***	0.115***	0.319***	0.363***	0.092**
	(0.040)	(0.050)	(0.038)	(0.040)	(0.050)	(0.038)
AT3 Calling orientation	− 0.258***	− 0.314***	− 0.032	− 0.076*	-0.110*	0.040
	(0.043)	(0.053)	(0.041)	(0.042)	(0.057)	(0.044)
*N*	1,526	1,526	1,228	1,454	1,454	1,217
Pseudo *R*^2^	0.257	0.159	0.125	0.339	0.222	0.147

Notes: The reported estimates are average marginal effects obtained after probit estimators. The individual controls in panel A are age and biological sex. The additional controls in panel B are marital status, children in the household, home ownership, college degree, personal income tertile, occupation, working hours, public employee status, and tenure. Panel C adds to the controls of panel B personality traits, and panel D adds job satisfaction in addition to all other controls. The reference category for work orientation is the “job” archetype (AT1). Robust standard errors in parentheses.

*p < 0.1, **p < 0.05, ***p < 0.01

#### Reverse causality

 We check whether work orientations predict outcomes beyond the following survey year. Because the orientations are measured before job separations occur, this analysis speaks to concerns that reported orientations merely determine contemporaneous job quit intentions and on-the-job search. Tables C2-C3 present descriptive statistics and estimation results for voluntary job separations occurring between 2023 and 2025, requiring respondents to be observed in both transition windows. Although the 2-year sample is slightly smaller and marginally older than the 1-year quit sample, it is otherwise similar in terms of demographic and occupational composition. The cumulative 2-year quit rate is 12%, compared to 7.5% over 1 year.

The estimation results in Table C3 are largely in line with the main findings. The career orientation remains a positive and statistically significant predictor of job separations across specifications, whereas job orientation remains unrelated to realized quits. Having a calling orientation is marginally negatively associated with job separations in parsimonious models, but loses statistical significance once additional controls are included.

#### Pre-existing exit plans

 In Table C4, we address the related concern that individuals who were already contemplating leaving may have adjusted their reported work orientations accordingly. To address this possibility, we control for prior job insecurity and prior quit expectations measured in the 2022 Work and Schooling Survey, i.e., before the work orientation module was fielded. These variables capture pre-existing exit plans and perceived risk of job loss.

The results remain largely unchanged. The career orientation continues to positively predict quit intentions, job search, and realized job quits, while having a stronger calling orientation remains negatively associated with intentions and search. The inclusion of prior quit expectations does not attenuate the main coefficients of interest. These findings suggest that work orientations do not merely reflect contemporaneous or anticipatory exit plans.

#### Including older workers

 In the main analysis, we exclude respondents aged 61 or older because their reported quit intentions and job search behavior may partly reflect retirement considerations rather than standard job mobility decisions. To assess whether this restriction drives our results, Table C5 re-estimates the main specifications including these older workers.

The results remain qualitatively unchanged. Career orientation continues to predict higher quit intentions, job search, and actual job quits across all specifications. The calling orientation remains negatively associated with quit intentions and job search, and is also negatively related to actual job quits in most models. The job orientation again shows no systematic association with any turnover outcome. Although the mean levels of quit intentions and job search are slightly lower in the expanded sample, the pattern of associations between work orientations and turnover outcomes closely mirrors the main results.

## Discussion and conclusion

This paper examines whether workers’ fundamental orientations toward work shape job search and quitting. We conceptualize work orientations as persistent beliefs about the role of work in one’s life, i.e., whether work is primarily a source of income (*job*), advancement (*career*), or fulfillment (*calling*). Using original survey data on work orientations from the Dutch LISS panel, we show that work orientations explain a substantial share of the variation in quit intentions, on-the-job-search, and, to a lesser extent, realized job quits, even after controlling for job satisfaction, socio-demographic characteristics, and personality traits. These findings are robust across alternative measurement approaches, including factor-based indices, categorical classifications, and archetypal profiles that allow for overlapping orientations.

Our findings reveal substantial heterogeneity in turnover behavior across workers. Having a job orientation is not significantly associated with mobility behavior. By contrast, a career orientation is positively associated with turnover-related behavior, particularly among workers who perceive limited promotion prospects. This pattern is consistent with tournament models of internal labor markets (Lazear 2018; Lazear & Oyer 2007), in which advancement opportunities shape incentives and mobility decisions. When upward mobility appears constrained, career-oriented workers are more likely to search for a new job and quit their current job.

Our findings regarding the calling orientation warrant nuance. While calling-oriented individuals exhibit lower quit intentions and job search on average, the interaction between having a calling orientation and perceived meaningfulness is subject to conceptual overlap. Moreover, because individuals may simultaneously hold multiple work orientations, it is plausible that compensatory mechanisms operate across motivational dimensions. For example, a calling-oriented worker who experiences low task-level meaningfulness may nevertheless remain in the job if it offers attractive career prospects or adequate compensation aligned with other orientations. Our results, therefore, do not imply that calling-oriented individuals are unconditionally attached to their jobs, but rather that their turnover behavior reflects the broader configuration of motivations and job attributes.

Our findings have implications for the world of work. The results underscore that employees differ systematically in their underlying motivations for working, and that these differences shape turnover-related behavior. Recognizing heterogeneity in work orientations may therefore improve the allocation of workers to jobs and reduce costly turnover arising from mismatches between individuals’ motivations and job attributes. For employers, the strong association between career orientation and turnover under limited promotion prospects suggests that transparent career pathways and advancement opportunities may be especially important for retaining career-oriented workers. For workers, awareness of one’s own work orientation may facilitate better job matching and career planning. Individuals who understand their orientation towards work may be better positioned to evaluate job offers, negotiate working conditions, and select employment environments consistent with their long-term motivations, with potential implications for job stability and sustained well-being over the life course.

We contribute to the literature in two main ways. First, we add to models of turnover and job search, which typically emphasize wages, job amenities, job satisfaction, and search frictions (Akerlof et al. 1988; Cornelißen 2009; Faberman & Justiniano 2015; Sullivan & To 2014), by showing that mobility decisions also depend on workers’ fundamental orientations toward work. Moreover, the results suggest that work orientations partly shape how workers respond to established determinants of mobility, including pay, promotion opportunities, and meaningful work (Clark 2001; Delfgaauw 2007; Cnossen & Lunardon 2026 ). Second, the findings connect to the literature on work meaning and non-monetary motivations (Burbano et al. 2023; Cassar & Meier 2018; Cotofan et al. 2023; Nikolova & Cnossen 2020) by identifying work orientations as a distinct and empirically relevant mechanism shaping turnover behavior. While prior research documents that workers value meaning, status, and autonomy, our results suggest that stable orientations toward work help explain why similar job characteristics translate into different mobility decisions across individuals.

This paper is a prototype analysis that we hope will inspire further investigations. Inevitably, it has limitations. First, work orientations are measured at a single point in time and in one institutional context (i.e., the Dutch), which precludes analysis of their long-term stability and cross-country variation. Although we link orientations to realized quits one and two years later, our estimates capture short-run associations rather than long-term career dynamics. Future longitudinal and cross-national data could assess the persistence and evolution of work orientations over the life course or across institutional environments.

Second, due to data limitations, our analysis focuses on workers and does not incorporate employer characteristics, management practices, or colleague-level effects, all of which may interact with workers’ orientations in shaping turnover behavior. Collecting relevant linked employer–employee data would allow researchers to examine how work orientations interact with organizational environments and whether matching between worker orientations and workplace characteristics affects retention, productivity, or wages.

Empirically, further work is needed to clarify the distinction between work orientations and related constructs such as work values or work ethic, and to test their stability across time and cultures. Emerging methods, including configurational approaches such as archetypal analysis, offer promising tools for capturing overlapping orientations. Finally, evolving forms of work, such as gig work and remote employment, may alter the salience and consequences of different work orientations and warrant systematic study.

## Supplementary Information

Below is the link to the electronic supplementary material.

ESM 1 Supplementary Material (PDF 1.11 MB)

ESM 2 Supplementary Material (ZIP 172 KB)

## Data Availability

All datasets listed below are available for download from the LISS data archive, using the links above. We have used Stata (version 19) to conduct the analyses. We relied on the archetypes package in R for archetypal analysis. Replication files are available on the Journal’s website. CenterData (2025). *Work and Schooling – Wave 18 (LISS Core Study)*, Project Number 6.18, https://doi.org/10.57990/e3mm-br41. CenterData (2024). The Great Resignation, Quiet Quitting, and Work Orientations, Project Number 350, https://doi.org/10.57990/pmq2-hq57. CenterData (2024). *Work and Schooling – Wave 17 (LISS Core Study)*, Project Number 6.17, https://doi.org/10.57990/e600-dp21. CenterData (2023). *Work and Schooling – Wave 16 (LISS Core Study)*, Project Number 6.16, https://doi.org/10.57990/vvb5-4c11. CenterData (2022). *Personality – Wave 14 (LISS Core Study)*, Project Number 7.15, https://doi.org/10.17026/dans-xwr-wwp3. CenterData (2022). *Work and Schooling – Wave 15 (LISS Core Study)*, Project Number 6.15, https://doi.org/10.17026/dans-x6u-sw4v.

## Footnotes

1 For example, a 10% increase in turnover is as costly as a 0.6% permanent increase in wages (Kuhn & Yu 2021). Moreover, a 1 SD increase in turnover leads to 1.6% of a standard deviation decrease in Return on Assets (ROA) (Li et al. 2022).

2 To assess potential non-response bias, we examined whether demographic or job-quality characteristics predict participation in our survey module. To this end, we merged information on the respondents who were selected to participate in the survey, provided by CenterData, to information on the respondents who ended up completing the questionnaires. The results in Table C1 (available in Online Supplementary Material) detail that respondents differ modestly from nonrespondents in age, gender, and parental status, but we observe no systematic differences in working conditions, including occupation, contract type, working hours, tenure, job satisfaction, job-quit risk, or perceived advancement opportunities. These patterns suggest that selective non-response is unlikely to distort the relationships analyzed in the paper.

3 Table C5 replicates our main analyses including the 303 older individuals with i.e., non-missing data, which we exclude in the main analyses. The main message of the paper remains the same.

4 We used the LISS-specific variable “profession” (cw23o404) as a proxy for occupation instead of the ISCO classification due to its fewer missing observations. We combined the "Basic Manual Labor" and "Agricultural Work" categories due to the small number of observations in each.

5 We also collected work orientations data using vignettes. However, only a minority of respondents consistently answered the vignette questions, suggesting that they may be too cognitively complex.

6 Analyses using the item Q7 about willingness to retire do not change the main conclusions in our paper. We show the scree plot in Fig. C1, the factor loadings are available in Table C8, and the main regression results replicating Table 3, are available in Table C9.

7 We assessed the measurement properties of the Work and Meaning Inventory (WAMI; Steger et al. 2012) using polychoric principal component analysis. The 10 items (Q14–Q23) yield three interpretable components: positive meaning (Q14–Q17), meaning-making through work (Q18–Q20), and greater-good motivation (Q21–Q23) (see Appendices A and B). These components account for 82%, 81%, and 89% of the variance in their respective item sets. Internal consistency is high across subscales (Cronbach’s α = 0.89, 0.85, and 0.92). The Kaiser–Meyer–Olkin measures indicate adequate sampling (KMO = 0.84, 0.70, and 0.73). We compute component scores for each subscale and sum them to form a composite index, which we rescale to range from 0 to 100 for ease of interpretation. The three sub-indices are moderately to strongly correlated (pairwise *r* = 0.60–0.64).

8 Estimating the models using logits or OLS does not alter the main conclusions.

9 There could also be selection into jobs based on work orientations. For example, career-oriented individuals may sort into high-mobility sectors and calling-oriented individuals may sort into mission-driven occupations. While we control for occupation, we cannot fully rule out sorting into firms with systematically different turnover dynamics.

10 The LISS panel includes two distinct measures on job satisfaction: one measured on a 0–10 scale and a separate agree–disagree item. Appendix Table C7 reports the full econometric output for both specifications. Job satisfaction is strongly and negatively associated with quit intentions, job search, and realized quits under both operationalizations. Importantly, the coefficients on career and calling orientations remain stable in magnitude and statistical significance across both measures. This robustness check indicates that work orientations capture dimensions of turnover-related behavior that are above and beyond job satisfaction.

11 We also examined whether the decomposition results depend on how job satisfaction is measured. Figure C2 reports the Shapley decomposition using the alternative agree–disagree job satisfaction item. The qualitative pattern remains unchanged. For quit intentions, work orientations account for approximately 50% of the explained variation, followed by job satisfaction (35%). For job search, orientations and job satisfaction contribute almost equally (both about 41%). For realized job quits, working conditions and job satisfaction each account for roughly 22% of the explained variation, while work orientations contribute 15%. Thus, although the relative importance of predictors shifts somewhat across outcomes, the central conclusion remains: work orientations are a major contributor to turnover intentions and search behavior and remain a meaningful determinant of realized quits.

12 Gen Z characterizes individuals born between 1997 and 2012. In our analysis sample, we have individuals aged as young as 18, so born in 2005. Millennials are those workers born between 1981 and 1996.

13 Archetype analysis has been adopted in several disciplines, ranging from economics and business (Beugelsdijk et al. 2022; De Wit and Beugelsdijk 2024; Venaik and Midgely 2015) to biology (Römer et al. 2012), and climatology (Hannachi and Trendafilov 2017; Steinschneider and Lall 2015).

14 The average value of Archetype 1 is 0.30 (SD 0.24), 0.29 for Archetype 2 (SD 0.23), and 0.40 for Archetype 3 (SD 0.21). The archetypal scores show a moderate to strong correlation with the three work orientation factors: AT1 correlates at 0.32 with job, AT2 correlates at.96 with career, and AT3 correlates at 0.92 with calling.
