# Supplementary material for: Work orientations and turnover
Source: J Popul Econ. 2026 Jun 6;39(3):33. doi: 10.1007/s00148-026-01176-w (PMC13242402; doi:10.1007/s00148-026-01176-w)
Supplement: Supplementary file 1 — Supplementary Material (PDF 1.11 MB) [file 148_2026_1176_MOESM1_ESM.pdf]

**SUPPLEMENTARY INFORMATION**

**Work Orientations and Turnover**

**Milena Nikolova and Juliette de Wit**

***Journal of Population Economics***

**May 6 2026**

**CONTENT**

**APPENDIX A – English language questionnaire** 2

**APPENDIX B – Dutch language questionnaire** 7

**APPENDIX C – Supplementary analyses** 12

**APPENDIX D – An archetypal typology of work orientations** 26

## APPENDIX A

## English language questionnaire (Translated from Dutch, see Online Appendix B)

### PART 1: WORK ORIENTATIONS

Below are three descriptions of categories of people. Please read all three carefully. For each category, indicate how well this category describes you.

**Category A** people work primarily to earn enough money to support their lives outside of their jobs. If they were financially secure, they would no longer continue with their current line of work, but would really rather do something else instead. To these people, their jobs are basically a necessity of life, a lot like breathing or sleeping. They often wish the time would pass more quickly at work. They greatly anticipate weekends and vacations. If these people lived their lives over again, they probably would not go into the same line of work. They would not encourage their friends and children to enter their line of work. Category A people are very eager to retire.

**Category B** people basically enjoy their work, but do not expect to be in their current jobs five years from now. Instead, they plan to move on to better, higher-level jobs. They have several goals for their futures pertaining to the positions they would eventually like to hold. Sometimes their work seems a waste of time, but they know that they must do sufficiently well in their current positions in order to move on. Category B people can't wait to get a promotion. For them, a promotion means recognition of their good work, and is a sign of their success in competition with coworkers.

For **Category C** people, work is one of the most important parts of life. They are very pleased that they are in their line of work. Because what they do for a living is a vital part of who they are, it is one of the first things they tell people about themselves. They tend to take their work home with them and on vacations, too. The majority of their friends are from their places of employment, and they belong to several organizations and clubs relating to their work. They feel good about their work because they love it, and because they think it makes the world a better place. They would encourage their friends and children to enter their line of work. Category C people would be pretty upset if they were forced to stop working, and they are not particularly looking forward to retirement.

How well does each of the above categories describe you?

*Question type:* Table
*Answer type:* Radio buttons
*Sub-questions:*
**Q1** Category A
**Q2** Category B
**Q3** Category C

*Categories*:
*1.* Not at all like me
*2.* Not really like me

*3.* A bit like me
*4.* Exactly like me

---

To what extent do the following statements about you, your work and/or career apply?

*Question type:* Table
*Answer type:* Radio buttons
*Sub-questions:*
**Q4** I enjoy talking about my work with others
**Q5** My work is one of the most important things in my life
**Q6** My main reason for working is financial: to support my family and lifestyle
**Q7** I am eager to retire
**Q8** If I was financially independent, I would continue my current work even if I wasn't getting paid for it
**Q9** My work makes the world a better place
**Q10** I would choose my current line of work again if we had the chance
**Q11** I expect to be in a higher-level job in five years
**Q12** I view my job as a stepping stone to other jobs
**Q13** I expect to be doing the same work in five years

*Categories*:
*1.* 1 Not applicable at all
*2.* 2
*3.* 3 Neutral
*4.* 4
*5.* 5 Completely applicable

---

**PART 2: WORK MEANINGFULNESS**

To what extent do the following statements about you, your work and/or career apply?

*Question type:* Table
*Answer type:* Radio buttons
*Sub-questions:*
**Q14** I have found a meaningful career
**Q15** I understand how my work contributes to my life's meaning
**Q16** I have a good sense of what makes my job meaningful
**Q17** I have discovered work that has a satisfying purpose
**Q18** I view my work as contributing to my personal growth
**Q19** My work helps me better understand myself
**Q20** My work helps me make sense of the world around me
**Q21** My work really makes a difference to the world
**Q22** I know my work makes a positive difference in the world
**Q23** The work I do serves a greater purpose

*Categories*:

1. 1 Not applicable at all
2. 2
3. 3 Neutral
4. 4
5. 5 Completely applicable

---

**PART 3: QUIT INTENTIONS**

**Q24** How likely is it that you will try to find a job with another firm or organization within the next 12 months?

*Answer type:* Radio buttons
*Categories:*
1. Very unlikely
2. Unlikely
3. Neither unlikely nor likely
4. Likely
5. Very likely

---

If Q24 > 2
**Q25** Why do you think you may no longer be working at your current job within the next 12 months? Choose the main reason.

*Answer type:* Radio buttons
*Categories:*
1. The organization/workplace will close down
2. I will be declared redundant
3. I will reach normal retirement age
4. My contract of employment will expire
5. I will take early retirement
6. I will decide to leave and work for another employer
7. I will decide to leave and work for myself as a self-employed
8. I will leave to look after the home/children/family
9. I will leave to participate in education/educational program
10. Running my own business is no longer financially worthwhile
11. Other, namely: ______________________________________ *(Answer type:* String*)*

---

If Q24 > 2
**Q26** What steps, if any, have you taken to find a job with another firm or organization?
*Multiple answers are possible.*

*Answer type:* Checkboxes
*Sub-questions*:
**Q26__1** I have updated my CV
**Q26__2** I have searched online job boards

**Q26__3** I have applied for job openings at other companies
**Q26__4** I have reached out to my professional network for job leads or recommendations
**Q26__5** I have attended job fairs or networking events
**Q26__6** I have contacted a recruiter or employment agency
**Q26__7** I have considered going back to school or pursuing additional training
**Q26__8** I have spoken with a career counselor or coach
**Q26__9** Other, namely: ______________________________ (*Answer type*: String)
**Q26__10** I have not taken any steps to find a job with another firm or organization

*Categories:*
*0.* No
*1.* Yes

---

**PART 4: QUIET QUITTING**

**Q27** How much effort are you currently putting into your main paid job?

*Answer type:* Slider
*Label left:* No effort 0%
*Label right:* A lot of effort 100%
*Min:* 0
*Max:* 100

---

Do you agree or disagree with the following statements:

*Question type:* Table
*Answer type*: Radio buttons
*Sub-questions:*
**Q28** Employees should always try to do their best at work
**Q29** Employees should set boundaries around the amount of extra work they do
**Q30** Employees should only do the work they are paid for, no more and no less

*Categories*:
*1.* Strongly disagree
*2.* Disagree
*3.* Neither agree nor disagree
*4.* Agree
*5.* Strongly agree

---

**Q31** Some employees do only the bare minimum of what they are asked to do to keep their jobs. They do not put in extra effort if there is no compensation in return. This phenomenon is called “quiet quitting.” How acceptable do you find it when someone does this?

*Answer type:* Radio buttons
*Categories:*
*1.* Not acceptable at all

*2.* Unacceptable
*3.* Neither unacceptable nor acceptable
*4.* Acceptable
*5.* Fully acceptable

# APPENDIX B

## Dutch language questionnaire

## Vragenlijst

### DEEL 1: WORK ORIENTATIONS

Hieronder staan drie omschrijvingen van groepen mensen. Leest u deze alle drie goed door. Geef voor elke groep aan hoe goed deze groep u omschrijft.

Mensen uit **groep A** werken vooral om voldoende geld te verdienen om te leven. Als ze financieel onafhankelijk zijn, zullen ze het werk niet blijven doen, maar liever iets anders gaan doen. Deze groep ziet een baan als iets dat nodig is om te leven, zoals ademen of slapen. Vaak hopen ze dat de tijd sneller gaat als ze aan het werk zijn. Ze kijken erg uit naar weekenden en vakanties. Als ze hun leven nog eens over mochten doen, zouden ze waarschijnlijk niet het werk doen dat ze nu doen. Ze moedigen hun vrienden en kinderen niet aan om hetzelfde werk te doen. Mensen uit groep A kunnen niet wachten om met pensioen te gaan.

Mensen uit **groep B** houden wel van hun werk, maar verwachten niet dat ze over vijf jaar nog hetzelfde werk doen. Ze zijn bezig om een betere baan op een hoger niveau te krijgen. Ze hebben verschillende doelen voor hun toekomst als het gaat om de functies die ze uiteindelijk willen bereiken. Hun werk lijkt soms tijdsverspilling, maar ze weten dat ze het redelijk goed moeten doen om hogerop te komen. Mensen uit groep B kunnen niet wachten om promotie te krijgen. Promotie is een erkenning dat ze hun werk goed doen, en een teken dat ze succesvol zijn in vergelijking met hun collega's.

Voor mensen uit **groep C** is werk één van de belangrijkste dingen in het leven. Ze zijn heel blij met het werk dat ze doen. Het is een wezenlijk deel van wie ze zijn, en het is daarom een van de eerste dingen waarover ze vertellen aan anderen. Ze hebben de neiging hun werk mee te nemen naar huis en op vakantie. Het grootste deel van hun vrienden hebben ze door hun werk verkregen. Ze zitten in verschillende organisaties en clubs die met hun werk te maken hebben. Ze voelen zich goed over hun werk omdat ze ervan houden, en omdat ze denken dat het nuttig is voor de maatschappij. Ze moedigen hun vrienden en kinderen aan om hetzelfde werk te gaan doen. Mensen uit groep C zouden overstuur zijn als ze moesten stoppen met werken, en ze kijken er niet echt naar uit om met pensioen te gaan.

Hoe goed beschrijft elke van de bovenstaande groepen u?

*Question type:* Table
*Answer type:* Radio buttons
*Sub-questions:*
**Q1** Groep A
**Q2** Groep B
**Q3** Groep C

*Categories*:
*5.* Helemaal niet zoals ik

*6.* Niet echt zoals ik
*7.* Een beetje zoals ik
*8.* Precies zoals ik

---

In welke mate zijn de volgende uitspraken over u, uw werk en/of carrière van toepassing?

*Question type:* Table
*Answer type:* Radio buttons
*Sub-questions:*
**Q4** Ik vind het leuk om met anderen over mijn werk te praten
**Q5** Mijn werk is één van de belangrijkste dingen in mijn leven
**Q6** Mijn belangrijkste reden om te werken is financieel, zodat ik kan zorgen voor mijn gezin en kan leven zoals ik leef
**Q7** Ik kijk ernaar uit om met pensioen te gaan
**Q8** Als ik financieel onafhankelijk was, zou ik blijven werken in de baan die ik nu heb, ook als ik er niet voor zou worden betaald
**Q9** Mijn werk verbetert de wereld
**Q10** Als ik de kans had, zou ik opnieuw kiezen voor het werk dat ik nu heb
**Q11** Ik verwacht over vijf jaar een baan te hebben op een hoger niveau
**Q12** Ik zie mijn baan als een opstap naar andere banen
**Q13** Ik verwacht over vijf jaar hetzelfde werk te doen als dat ik nu doe

*Categories*:
*6.* 1 Helemaal niet van toepassing
*7.* 2
*8.* 3 Neutraal
*9.* 4
*10.* 5 Helemaal van toepassing

---

## DEEL 2: WORK MEANINGFULNESS

In welke mate zijn de volgende uitspraken op u, uw werk en/of carrière van toepassing?

*Question type:* Table
*Answer type:* Radio buttons
*Sub-questions:*
**Q14** Ik heb een zinvolle carrière gevonden
**Q15** Ik begrijp hoe mijn werk bijdraagt aan het doel van mijn leven
**Q16** Ik heb een goed idee bij wat mijn werk zinvol maakt
**Q17** Ik heb werk gevonden dat een bevredigend doel heeft
**Q18** Ik zie mijn werk als een bijdrage aan mijn persoonlijke groei
**Q19** Mijn werk helpt mij mezelf beter te begrijpen
**Q20** Mijn werk helpt mij om de wereld om mij heen beter te begrijpen
**Q21** Mijn werk maakt echt een verschil in de wereld
**Q22** Ik weet dat mijn werk een positief verschil maakt in de wereld
**Q23** Het werk dat ik doe dient een groter doel

*Categories*:

6. 1 Helemaal niet van toepassing
7. 2
8. 3 Neutraal
9. 4
10. 5 Helemaal van toepassing

---

**DEEL 3: QUIT INTENTIONS**

**Q24** Hoe waarschijnlijk is het dat u in de komende 12 maanden een baan gaat zoeken bij een ander bedrijf of een andere organisatie?

*Answer type:* Radio buttons
*Categories:*

6. Erg onwaarschijnlijk
7. Onwaarschijnlijk
8. Niet onwaarschijnlijk en niet waarschijnlijk
9. Waarschijnlijk
10. Erg waarschijnlijk

---

If Q24 > 2
**Q25** Waarom denkt u dat u over 12 maanden misschien niet meer werkt in de baan die u op dit moment hebt? Kies de belangrijkste reden.

*Answer type:* Radio buttons
*Categories:*

12. De organisatie/werkplek gaat sluiten
13. Ik zal overbodig zijn
14. Ik bereik de pensioengerechtigde leeftijd
15. Mijn arbeidsovereenkomst loopt af
16. Ik ga met vervroegd pensioen
17. Ik zal besluiten om te vertrekken om voor een andere werkgever te gaan werken
18. Ik zal besluiten om te vertrekken om voor mijzelf te gaan werken als zelfstandige
19. Ik zal vertrekken om voor huis/kinderen/familielid te zorgen
20. Ik zal vertrekken om een opleiding/cursus te volgen
21. Een eigen bedrijf hebben is financieel niet meer de moeite waard
22. Anders, namelijk: ______________________________________ *(Answer type:* String*)*

---

If Q24 > 2
**Q26** Wat hebt u eventueel gedaan om een baan te vinden bij een ander bedrijf of een andere organisatie?
*Meerdere antwoorden mogelijk*

*Answer type:* Checkboxes

*Subquestions*:
**Q26__1** Ik heb mijn CV bijgewerkt
**Q26__2** Ik heb op online vacaturesites gezocht
**Q26__3** Ik heb gesolliciteerd op vacatures bij andere bedrijven
**Q26__4** Ik heb contact gezocht met mijn professionele netwerk voor vacatures of aanbevelingen
**Q26__5** Ik heb banenbeurzen of netwerkevenementen bezocht
**Q26__6** Ik heb contact gehad/gezocht met een recruiter of uitzendbureau
**Q26__7** Ik heb erover nagedacht weer terug naar school te gaan of een aanvullende opleiding te volgen
**Q26__8** Ik heb met een loopbaanadviseur of -coach gesproken
**Q26__9** Anders, namelijk: ______________________________ (*Answer type*: String)
**Q26__10** Ik heb niets gedaan om een baan te vinden bij een ander bedrijf of andere organisatie

*Categories:*
*2.* Nee
*3.* Ja

---

**DEEL 4: QUIET QUITTING**

**Q27** Hoeveel moeite doet u op dit moment in uw belangrijkste betaalde baan?
*Answer type:* Slider
*Label left:* Geen moeite 0%
*Label right:* Zeer veel moeite 100%
*Min:* 0
*Max:* 100

---

Bent u het oneens of eens met de volgende uitspraken?

*Question type:* Table
*Answer type*: Radio buttons
*Sub-questions:*
**Q28** Werknemers moeten altijd proberen hun uiterste best te doen
**Q29** Werknemers moeten grenzen stellen aan de hoeveelheid extra werk dat ze doen
**Q30** Werknemers moeten alleen het werk doen waarvoor ze betaald krijgen, niet meer en niet minder

*Categories*:
*6.* Helemaal niet mee eens
*7.* Niet mee eens
*8.* Niet eens, niet oneens
*9.* Mee eens
*10.* Helemaal mee eens

---

**Q31** Sommige medewerkers van een bedrijf doen alleen het minimale van wat ze gevraagd wordt om hun baan te behouden. Ze spannen zich niet extra in als daar geen compensatie tegenover staat. Dit wordt ‘quiet quitting’ of ‘stil stoppen’ genoemd. Hoe acceptabel vindt u het als iemand dit doet?

*Answer type:* Radio buttons
*Categories:*

6. Helemaal niet acceptabel
7. Niet acceptabel
8. Niet onacceptabel en niet acceptabel
9. Acceptabel
10. Volledig acceptabel

## APPENDIX C

## Supplementary Analyses

Table C1: Selection into non-response for the survey module

| | Responded to survey invitation (1) | Responded to survey invitation (2) |
|---|---|---|
| Age | -0.010** | 0.002 |
| | (0.005) | (0.003) |
| $Age^2/100$ | 0.019*** | 0.000 |
| | (0.006) | (0.003) |
| Male | -0.037** | -0.017* |
| | (0.016) | (0.009) |
| Gender: other | 0.062 | 0.143 |
| | (0.160) | (0.092) |
| Married | 0.022 | -0.001 |
| | (0.017) | (0.009) |
| Children | -0.038** | -0.017* |
| | (0.018) | (0.010) |
| Household size | 0.028 | 0.020* |
| | (0.024) | (0.012) |
| Homeowner | -0.004 | -0.004 |
| | (0.018) | (0.010) |
| Urban | -0.005 | -0.003 |
| | (0.015) | (0.008) |
| Urban residence: missing information | -0.073 | 0.014 |
| | (0.176) | (0.033) |
| Higher education | -0.003 | 0.011 |
| | (0.017) | (0.008) |
| Higher education missing | 0.009 | 0.070 |
| | (0.093) | (0.053) |
| Middle income | 0.014 | -0.011 |
| | (0.019) | (0.011) |
| High income | -0.022 | -0.026** |
| | (0.022) | (0.011) |
| Income missing | 0.044 | -0.013 |
| | (0.033) | (0.020) |
| Supervisory | | 0.130** |
| | | (0.060) |
| Intermediate academic | | 0.029 |
| | | (0.036) |
| Intermediate supervisory | | -0.009 |
| | | (0.040) |
| Other intermediate | | 0.071 |
| | | (0.044) |
| Skilled manual | | 0.094 |
| | | (0.067) |
| Semi-skilled | | 0.092 |

| | | |
|---|---|---|
| | | (0.070) |
| Unskilled | | 0.076 |
| | | (0.078) |
| Profession missing | | -0.165 |
| | | (0.175) |
| Temporary contract | | -0.009 |
| | | (0.011) |
| Self-employed | | -0.023 |
| | | (0.031) |
| Contract type missing | | -0.940*** |
| | | (0.004) |
| Middle tertile working hours | | -0.006 |
| | | (0.005) |
| Top tertile working hours | | 0.004 |
| | | (0.007) |
| Working hours missing | | 0.059*** |
| | | (0.005) |
| Tenure: 2-5 years in company | | -0.016 |
| | | (0.034) |
| Tenure: 6+ years in company | | -0.031 |
| | | (0.033) |
| Missing tenure | | -0.094 |
| | | (0.064) |
| Job quit chance >=50% | | 0.009 |
| | | (0.046) |
| Job quit chance missing | | -0.037 |
| | | (0.023) |
| Job satisfaction: disagree | | 0.038 |
| | | (0.084) |
| Job satisfaction: agree | | 0.051 |
| | | (0.077) |
| Job satisfaction: agree entirely | | -0.028 |
| | | (0.074) |
| Missing job satisfaction | | -0.044 |
| | | (0.142) |
| No career advancement | | -0.009 |
| | | (0.013) |
| Career advancement missing | | 0.035 |
| | | (0.035) |
| N | 3,421 | 3,421 |
| Pseudo $R^2$ | 0.674 | 0.674 |

Notes: Reference categories: female, non-urban residence, no higher education, low income, higher academic profession, permanent employment, lowest working-hours tertile, tenure ≤ 1 year, job-quit chance < 50%, job satisfaction “disagree entirely,” and career advancement possible. The dependent variable equals 1 if a selected respondent completed the survey (N respondents = 2,525). CenterData initially sampled 3,428 LISS panel members. After excluding five non-working individuals (one job seeker, one student, two pensioners, and one volunteer), 3,423 respondents were eligible. The estimation sample equals 3,421 due to two missing values on home ownership (the category “owner = missing” predicts nonresponse perfectly, so Stata automatically drops these cases). Robust standard errors in parentheses. * p<0.1, ** p<0.05, *** p<0.01.

Table C2: Selected summary statistics, analysis sample with job separations between 2023 and 2025

| | Job Quits Between 2023-2025 Sample, N=1,096 | |
|---|---|---|
| Variable | Mean | Std. Dev. |
| Age | 45.205 | 10.585 |
| Male | 0.495 | 0.500 |
| Married | 0.520 | 0.500 |
| One or more children | 0.512 | 0.500 |
| Owner | 0.779 | 0.415 |
| Higher education | | |
| No | 0.480 | 0.500 |
| Yes (WO and HBO) | 0.516 | 0.500 |
| No information | 0.004 | 0.060 |
| Personal net income tertile | | |
| Poorest | 0.266 | 0.442 |
| Middle | 0.336 | 0.472 |
| Richest | 0.343 | 0.475 |
| No information | 0.055 | 0.228 |
| Profession | | |
| Advanced Academic/Professional (Architect, Physician, Scholar) | 0.141 | 0.348 |
| Senior Management (Manager, Director, Company Owner) | 0.091 | 0.288 |
| Intermediate Professional (Teacher, Artist, Nurse) | 0.269 | 0.444 |
| Mid-Level Supervisory/Commercial (Department Manager, Shopkeeper) | 0.108 | 0.310 |
| Clerical and Support Work (Administrative Assistant, Accountant) | 0.199 | 0.399 |
| Skilled Manual Work (Car Mechanic, Foreman) | 0.057 | 0.233 |
| Semi-Skilled Manual Work (Driver, Factory Worker) | 0.066 | 0.248 |
| Basic Manual Labor (Cleaner, Packer) | 0.037 | 0.190 |
| Missing information | 0.032 | 0.176 |

Table C3: The relationship between work orientations and job separations, 2023-2025

| | Job separations (1) | Job separations (2) | Job separations (3) | Job separations (4) |
|---|---|---|---|---|
| Job orientation | -0.003 | 0.000 | 0.000 | -0.006 |
| | (0.011) | (0.010) | (0.011) | (0.011) |
| Career orientation | 0.044*** | 0.043*** | 0.042*** | 0.034*** |
| | (0.011) | (0.011) | (0.011) | (0.012) |
| Calling orientation | -0.019* | -0.017* | -0.022** | -0.013 |
| | (0.010) | (0.010) | (0.010) | (0.012) |
| Controls | Exogenous individual | All individual controls | All individual controls + personality traits | All individual controls + personality traits + job satisfaction |
| N | 1,096 | 1,096 | 1,037 | 1,009 |
| Mean DV | 0.120 | 0.120 | 0.119 | 0.119 |
| Pseudo $R^2$ | 0.055 | 0.126 | 0.140 | 0.148 |

Notes: The reported estimates are average marginal effects obtained after probit estimators. The dependent variable is a binary indicator equal to 1 if the respondent voluntarily changed jobs either between 2023–2024 or between 2024–2025, conditional on being observed in both periods; 0 otherwise. The individual controls in (1) are age and gender. The additional controls in (2) are marital status, children in the household, home ownership, college degree, personal income tertile, occupation, working hours, public employee status, and tenure. (3) adds to the controls of Panel B personality traits, and (4) adds job satisfaction in addition to all other controls. The work orientations indices are standardized to have a mean of 0 and a standard deviation of 1. Summary statistics for this sample are available in Table C2. Robust standard errors in parentheses. * p<0.1, ** p<0.05, *** p<0.01.

Table C4: The relationship between work orientations and quit intentions, job search, and job quits, with controls for job loss and job quits in t-1

| | Quit intention (1) | Quit intention (2) | Job search (3) | Job search (4) | Job quit (5) | Job quit (6) |
|---|---|---|---|---|---|---|
| Job orientation | -0.000 | -0.000 | -0.006 | -0.006 | 0.004 | 0.005 |
| | (0.010) | (0.010) | (0.013) | (0.013) | (0.010) | (0.010) |
| Career orientation | 0.091*** | 0.092*** | 0.107*** | 0.105*** | 0.032*** | 0.028*** |
| | (0.010) | (0.011) | (0.013) | (0.013) | (0.010) | (0.010) |
| Calling orientation | -0.047*** | -0.053*** | -0.067*** | -0.077*** | 0.003 | -0.001 |
| | (0.009) | (0.009) | (0.012) | (0.012) | (0.009) | (0.009) |
| Job loss chance in t-1 | -0.002 | 0.001 | -0.020 | -0.013 | 0.015* | 0.011 |
| | (0.008) | (0.008) | (0.012) | (0.012) | (0.008) | (0.007) |
| Job quit chance in t-1 | 0.039*** | 0.040*** | 0.053*** | 0.065*** | 0.020** | 0.017** |
| | (0.008) | (0.008) | (0.012) | (0.012) | (0.008) | (0.008) |
| Controls | Exogenous | Full | Exogenous | Full | Exogenous | Full |
| N | 981 | 979 | 981 | 979 | 819 | 805 |
| Mean DV | 0.111 | 0.111 | 0.209 | 0.209 | 0.073 | 0.075 |
| Pseudo $R^2$ | 0.278 | 0.303 | 0.152 | 0.206 | 0.100 | 0.184 |

Notes: The reported estimates are average marginal effects obtained after probit estimation. The exogenous controls include age and gender. The full set of controls additionally includes marital status, children in the household, home ownership, college degree, personal income tertile, occupation, working hours, public employee status, and tenure. Some specifications further control for personality traits and job satisfaction. Job loss chance and job quit chance are measured in the 2022 Work and Schooling Survey and are standardized to have a mean of 0 and a standard deviation of 1. The work orientation indices are standardized to have a mean of 0 and a standard deviation of 1. Robust standard errors in parentheses. * p<0.1, ** p<0.05, *** p<0.01.

Table C5: The relationship between work orientations and quit intentions, job search, and job quits, with workers 61-67 included

| | Quit intention (1) | Job search (2) | Job quit (3) | Quit intention (4) | Job search (5) | Job quit (6) |
|---|---|---|---|---|---|---|
| | Panel A: Exogenous Individual Controls | | | Panel B: All Individual Controls | | |
| Job orientation | -0.000 | -0.006 | -0.013 | 0.002 | -0.001 | -0.012 |
| | (0.006) | (0.008) | (0.008) | (0.006) | (0.008) | (0.008) |
| Career orientation | 0.079*** | 0.095*** | 0.041*** | 0.078*** | 0.094*** | 0.039*** |
| | (0.007) | (0.008) | (0.008) | (0.007) | (0.009) | (0.008) |
| Calling orientation | -0.064*** | -0.078*** | -0.020*** | -0.070*** | -0.083*** | -0.022*** |
| | (0.006) | (0.008) | (0.008) | (0.006) | (0.008) | (0.008) |
| N | 2,051 | 2,051 | 1,598 | 2,051 | 2,051 | 1,578 |
| Mean DV | 0.0956 | 0.175 | 0.0870 | 0.0956 | 0.175 | 0.0881 |
| Pseudo $R^2$ | 0.238 | 0.148 | 0.0364 | 0.261 | 0.166 | 0.0769 |
| | Panel C: All Individual Controls + Personality Traits | | | Panel D: All Individual Controls + Personality + Job satisfaction | | |
| Job orientation | -0.000 | -0.003 | -0.012 | 0.001 | -0.001 | -0.014* |
| | (0.007) | (0.009) | (0.008) | (0.007) | (0.008) | (0.008) |
| Career orientation | 0.084*** | 0.091*** | 0.038*** | 0.071*** | 0.079*** | 0.033*** |
| | (0.008) | (0.010) | (0.009) | (0.008) | (0.010) | (0.009) |
| Calling orientation | -0.069*** | -0.085*** | -0.022*** | -0.036*** | -0.043*** | -0.012 |
| | (0.007) | (0.009) | (0.008) | (0.007) | (0.010) | (0.009) |
| N | 1,808 | 1,808 | 1,467 | 1,729 | 1,729 | 1,456 |
| Mean DV | 0.0957 | 0.174 | 0.087 | 0.093 | 0.168 | 0.087 |
| Pseudo $R^2$ | 0.286 | 0.180 | 0.093 | 0.352 | 0.231 | 0.101 |

Notes: The reported estimates are average marginal effects obtained after probit estimators. The individual controls in Panel A are age and gender. The additional controls in Panel B are marital status, children in the household, home ownership, college degree, personal income tertile, occupation, working hours, public employee status, and tenure. Panel C adds to the controls of Panel B personality traits, and Panel D adds job satisfaction in addition to all other controls. The work orientations indices are standardized to have a mean of 0 and a standard deviation of 1. Robust standard errors in parentheses. * p<0.1, ** p<0.05, *** p<0.01.

Table C6: Correlation between job satisfaction questions and work orientation items

| | 1. | 2. | 3. | 4. | 5. | 6. | 7. | 8. | 9. | 11. | 12. |
|---|---|---|---|---|---|---|---|---|---|---|---|
| 1. Job satisfaction - 10 point | 1 | | | | | | | | | | |
| 2. Job satisfaction - agree-disagree | 0.663 | 1 | | | | | | | | | |
| 3. Q4 I enjoy talking about my work with others | 0.339 | 0.277 | 1 | | | | | | | | |
| 4. Q5 My work is one of the most important things in my life | 0.302 | 0.273 | 0.486 | 1 | | | | | | | |
| 5. Q6 My main reason for working is financial: to support my family and lifestyle | -0.139 | -0.128 | -0.143 | -0.201 | 1 | | | | | | |
| 6. Q8 If I was financially independent, I would continue my current work even if I wasn't getting paid for it | 0.284 | 0.271 | 0.297 | 0.369 | -0.320 | 1 | | | | | |
| 7. Q9 My work makes the world a better place | 0.219 | 0.181 | 0.346 | 0.277 | -0.144 | 0.299 | 1 | | | | |
| 8. Q10 I would choose my current line of work again if I had the chance | 0.478 | 0.419 | 0.390 | 0.385 | -0.206 | 0.403 | 0.407 | 1 | | | |
| 9. Q11 I expect to be in a higher-level job in five years | -0.081 | -0.049 | 0.155 | 0.100 | -0.030 | 0.080 | 0.154 | 0.069 | 1 | | |
| 10. Q12 I view my job as a stepping stone to other jobs | -0.145 | -0.140 | 0.056 | 0.039 | 0.040 | 0.016 | 0.092 | -0.043 | 0.738 | 1 | |
| 11. Q13 I expect to be doing the same work in five years (reversed) | -0.343 | -0.283 | -0.103 | -0.180 | 0.046 | -0.184 | -0.066 | -0.296 | 0.480 | 0.494 | 1 |

Notes: N=1,544 (i.e., all available observations). All variables are standardized to have a mean of 0 and standard deviation of 1.

Table C7: Work orientations and job satisfaction (full results underlying Shapley Decomposition in Figure 5)

| | Quit intention (1) | Job search (2) | Job quit (3) | Quit intention (4) | Job search (5) | Job quit (6) |
|---|---|---|---|---|---|---|
| Job orientation (std) | 0.006 | -0.002 | -0.004 | 0.006 | 0.001 | -0.003 |
| | (0.008) | (0.010) | (0.008) | (0.008) | (0.010) | (0.008) |
| Career orientation (std) | 0.084*** | 0.097*** | 0.022*** | 0.083*** | 0.095*** | 0.021** |
| | (0.009) | (0.011) | (0.008) | (0.009) | (0.011) | (0.008) |
| Calling orientation (std) | -0.028*** | -0.041*** | 0.004 | -0.033*** | -0.037*** | 0.006 |
| | (0.008) | (0.011) | (0.009) | (0.008) | (0.012) | (0.008) |
| Job satisfaction (0-10 scale, standardized) | -0.061*** | -0.087*** | -0.027*** | | | |
| | (0.007) | (0.011) | (0.008) | | | |
| Job satisfaction (agree-disagree, standardized) | | | | -0.067*** | -0.102*** | -0.034*** |
| | | | | (0.009) | (0.012) | (0.008) |
| Age | 0.001 | 0.000 | -0.000 | 0.000 | -0.001 | -0.000 |
| | (0.001) | (0.001) | (0.001) | (0.001) | (0.001) | (0.001) |
| Male | -0.019 | -0.040* | -0.026 | -0.018 | -0.037* | -0.030* |
| | (0.016) | (0.022) | (0.018) | (0.016) | (0.022) | (0.017) |
| Married | 0.016 | 0.025 | 0.017 | 0.010 | 0.029 | 0.018 |
| | (0.014) | (0.019) | (0.016) | (0.015) | (0.020) | (0.016) |
| One or more children | -0.007 | -0.024 | 0.013 | -0.009 | -0.031 | 0.015 |
| | (0.014) | (0.019) | (0.015) | (0.014) | (0.019) | (0.015) |
| Home owner | 0.003 | -0.012 | -0.009 | 0.010 | -0.008 | -0.006 |
| | (0.016) | (0.024) | (0.018) | (0.016) | (0.023) | (0.017) |
| Higher education (ref: no higher education) | | | | | | |
| Higher education (WO and HBO) | 0.007 | -0.004 | 0.010 | 0.009 | 0.008 | 0.013 |
| | (0.017) | (0.023) | (0.018) | (0.017) | (0.023) | (0.017) |
| No information | -0.017 | 0.125 | 0.106 | -0.024 | 0.044 | 0.044 |
| | (0.071) | (0.139) | (0.146) | (0.078) | (0.137) | (0.087) |
| Personal net income tertile (ref: poorest) | | | | | | |
| Middle | 0.015 | 0.001 | 0.014 | 0.020 | -0.003 | 0.014 |
| | (0.018) | (0.026) | (0.022) | (0.018) | (0.027) | (0.023) |
| Richest | 0.014 | 0.021 | -0.031 | 0.017 | 0.019 | -0.036* |
| | (0.022) | (0.030) | (0.021) | (0.022) | (0.031) | (0.020) |
| No information | 0.038 | 0.085* | 0.031 | 0.030 | 0.086* | 0.013 |
| | (0.034) | (0.049) | (0.037) | (0.031) | (0.048) | (0.034) |
| Profession (ref: advanced academic/professional (architect, physician, scholar) | | | | | | |
| Senior Management (Manager, Director, Company Owner) | 0.001 | -0.022 | 0.026 | 0.009 | -0.005 | 0.025 |
| | (0.029) | (0.039) | (0.033) | (0.029) | (0.041) | (0.033) |
| Intermediate Professional (Teacher, Artist, Nurse) | 0.034 | -0.003 | 0.029 | 0.029 | -0.003 | 0.021 |
| | (0.024) | (0.033) | (0.027) | (0.023) | (0.032) | (0.027) |
| Mid-Level Supervisory/Commercial (Department Manager, Shopkeeper) | -0.013 | -0.059 | -0.011 | -0.008 | -0.045 | -0.009 |
| | (0.024) | (0.036) | (0.027) | (0.024) | (0.037) | (0.028) |
| Clerical and Support Work (Administrative Assistant, Accountant) | -0.002 | -0.040 | -0.026 | 0.015 | -0.017 | -0.020 |
| | (0.024) | (0.036) | (0.025) | (0.025) | (0.036) | (0.026) |
| Skilled Manual Work (Car Mechanic, Foreman) | 0.023 | -0.093** | -0.028 | 0.014 | -0.101** | -0.036 |
| | (0.042) | (0.047) | (0.036) | (0.040) | (0.043) | (0.033) |

| | | | | | | |
|---|---|---|---|---|---|---|
| Semi-Skilled Manual Work (Driver, Factory Worker) | -0.051* | -0.068 | -0.004 | -0.044 | -0.060 | -0.004 |
| | (0.031) | (0.048) | (0.038) | (0.031) | (0.048) | (0.038) |
| Basic Manual Labor (Cleaner, Packer) | -0.055** | -0.094* | 0.002 | -0.050* | -0.082 | -0.003 |
| | (0.028) | (0.050) | (0.043) | (0.029) | (0.050) | (0.042) |
| No information | 0.030 | 0.017 | 0.072 | | | |
| | (0.045) | (0.060) | (0.061) | | | |
| Working hours tertile (ref: lowest) | | | | | | |
| Middle | -0.014 | -0.013 | -0.023 | -0.016 | -0.021 | -0.019 |
| | (0.018) | (0.024) | (0.018) | (0.019) | (0.024) | (0.018) |
| Top | -0.005 | 0.006 | -0.044** | -0.010 | -0.011 | -0.047** |
| | (0.025) | (0.033) | (0.021) | (0.025) | (0.032) | (0.020) |
| Public sector (ref: no) | | | | | | |
| Yes | 0.003 | -0.005 | -0.036** | 0.011 | -0.000 | -0.035** |
| | (0.015) | (0.020) | (0.014) | (0.015) | (0.020) | (0.014) |
| No information | 0.041 | -0.000 | | 0.038 | 0.014 | |
| | (0.081) | (0.091) | | (0.080) | (0.086) | |
| Tenure (ref: 0-1 years) | | | | | | |
| 2-5 years | -0.033 | 0.071** | -0.018 | -0.030 | 0.081*** | -0.008 |
| | (0.022) | (0.028) | (0.026) | (0.023) | (0.028) | (0.026) |
| 6+ years | -0.036* | 0.039 | -0.056** | -0.052** | 0.030 | -0.053** |
| | (0.021) | (0.026) | (0.025) | (0.022) | (0.027) | (0.025) |
| No information | | | | | 0.077 | |
| | | | | | (0.226) | |
| Extraversion | -0.009 | -0.010 | -0.013 | -0.012 | -0.014 | -0.018** |
| | (0.008) | (0.010) | (0.008) | (0.007) | (0.010) | (0.008) |
| Agreeableness | 0.013 | 0.005 | -0.004 | 0.008 | -0.004 | -0.007 |
| | (0.009) | (0.011) | (0.008) | (0.009) | (0.011) | (0.008) |
| Conscientiousness | 0.001 | 0.013 | -0.002 | -0.000 | 0.009 | -0.002 |
| | (0.007) | (0.010) | (0.007) | (0.007) | (0.010) | (0.007) |
| Emotional stability | -0.001 | 0.004 | 0.004 | -0.002 | 0.006 | 0.006 |
| | (0.007) | (0.010) | (0.008) | (0.007) | (0.010) | (0.008) |
| Imagination | 0.001 | 0.013 | 0.025*** | 0.001 | 0.012 | 0.025*** |
| | (0.008) | (0.011) | (0.008) | (0.009) | (0.011) | (0.008) |
| Observations | 1,454 | 1,454 | 1,217 | 1,413 | 1,417 | 1,192 |
| Mean DV | 0.108 | 0.195 | 0.075 | 0.105 | 0.194 | 0.072 |
| Pseudo $R^2$ | 0.344 | 0.226 | 0.149 | 0.343 | 0.235 | 0.156 |

Notes: The reported estimates are average marginal effects obtained after probit estimators. Models (1)-(3) provide full econometric output for Table 3, Panel D. The job satisfaction variable there is originally measured on a 0-10 Likert scale. Models (4)-(6) use an alternative job satisfaction variable measured on a 1-4 agree-disagree scale. The work orientations indices, job satisfaction variables, and the personality traits are standardized to have a mean of 0 and a standard deviation of 1. Robust standard errors in parentheses. * p<0.1, ** p<0.05, *** p<0.01.

Table C8: Factor analysis of work orientation items: varimax-rotated component loadings, with the item “I am eager to retire”

| | Component 1 (Calling) | Component 2 (Career) | Component 3 (job) |
|---|---|---|---|
| Q4 I enjoy talking about my work with others | 0.479 | 0.062 | 0.075 |
| Q5 My work is one of the most important things in my life | 0.428 | -0.006 | -0.049 |
| Q6 My main reason for working is financial: to support my family and lifestyle | 0.064 | 0.042 | 0.706 |
| Q7 I am eager to retire | -0.034 | -0.082 | 0.603 |
| Q8 If I was financially independent, I would continue my current work even if I wasn't getting paid for it | 0.305 | -0.035 | -0.330 |
| Q9 My work makes the world a better place | 0.454 | 0.096 | 0.129 |
| Q10 I would choose my current line of work again if we had the chance | 0.468 | -0.081 | -0.024 |
| Q11 I expect to be in a higher-level job in five years | 0.108 | 0.591 | -0.007 |
| Q12 I view my job as a stepping stone to other jobs | 0.038 | 0.601 | 0.034 |
| Q13 I expect to be doing the same work in five years (reversed) | -0.224 | 0.510 | -0.055 |

Notes: N=1,748. We standardized all items before applying polychoric principal component analysis. The main difference with Table 1 is that we here include the item Q7 “I am eager to retire.” After varimax rotation, the eigenvalues of the three retained components are 2.74, 2.28, and 1.17, explaining 30.5%, 25.4%, and 13.0% of the total variance, respectively. The three-factor solution explains 69% of the total variance in the included items.

Table C9: The relationship between work orientations and quit intentions, job search, and job quits, including item "I am eager to retire"

| | Quit intention (1) | Job search (2) | Job quit (3) | Quit intention (4) | Job search (5) | Job quit (6) |
|---|---|---|---|---|---|---|
| | Panel A: Exogenous Individual Controls | | | Panel B: All Individual Controls | | |
| Job orientation | 0.009 | 0.007 | 0.006 | 0.011 | 0.011 | 0.004 |
| | (0.007) | (0.010) | (0.008) | (0.008) | (0.010) | (0.008) |
| Career orientation | 0.098*** | 0.119*** | 0.035*** | 0.097*** | 0.119*** | 0.032*** |
| | (0.008) | (0.009) | (0.008) | (0.008) | (0.010) | (0.008) |
| Calling orientation | -0.058*** | -0.071*** | -0.005 | -0.064*** | -0.076*** | -0.007 |
| | (0.007) | (0.009) | (0.008) | (0.007) | (0.010) | (0.008) |
| N | 1,748 | 1,748 | 1,346 | 1,748 | 1,748 | 1,328 |
| Mean DV | 0.110 | 0.200 | 0.075 | 0.110 | 0.200 | 0.076 |
| Pseudo $R^2$ | 0.231 | 0.137 | 0.057 | 0.252 | 0.158 | 0.108 |
| | Panel C: All Individual Controls + Personality Traits | | | Panel D: All Individual Controls + Personality + Job satisfaction | | |
| Job orientation | 0.007 | 0.012 | 0.003 | 0.003 | 0.008 | -0.001 |
| | (0.008) | (0.010) | (0.008) | (0.008) | (0.010) | (0.008) |
| Career orientation | 0.106*** | 0.117*** | 0.031*** | 0.086*** | 0.096*** | 0.022** |
| | (0.009) | (0.011) | (0.008) | (0.009) | (0.011) | (0.008) |
| Calling orientation | -0.062*** | -0.079*** | -0.009 | -0.028*** | -0.038*** | 0.005 |
| | (0.008) | (0.010) | (0.008) | (0.008) | (0.011) | (0.009) |
| N | 1,526 | 1,526 | 1,228 | 1,454 | 1,454 | 1,217 |
| Mean DV | 0.111 | 0.201 | 0.0749 | 0.108 | 0.195 | 0.075 |
| Pseudo $R^2$ | 0.275 | 0.170 | 0.128 | 0.344 | 0.225 | 0.148 |

Notes: This is a replication of Table 3 in the main text but including the item "I am willing to retire." The reported estimates are average marginal effects obtained after probit estimators. The individual controls in Panel A are age and gender. The additional controls in Panel B are marital status, children in the household, home ownership, college degree, personal income tertile, occupation, working hours, public employee status, and tenure. Panel C adds to the controls of Panel B personality traits, and Panel D adds job satisfaction in addition to all other controls. The work orientations indices are standardized to have a mean of 0 and a standard deviation of 1. Robust standard errors in parentheses. * p<0.1, ** p<0.05, *** p<0.01.

Figure C1: Scree Plot, Work Orientations, Including Item "I Am Eager To Retire"

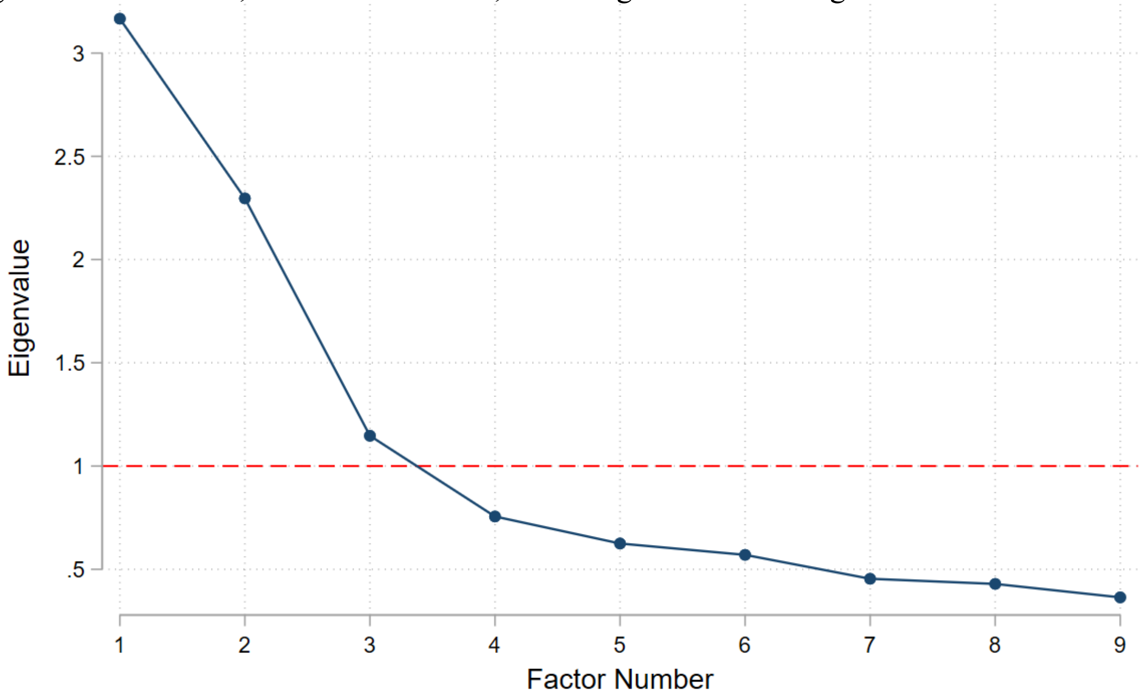


Note: Notes: N=1,748. We standardized all items before applying polychoric principal component analysis. The main difference with Figure 1 is that this scree plot includes the item Q7 "I am eager to retire."

Figure C2: Relative Contribution of Variable Groups to Explained Variance in Turnover Outcomes, Alternative Job Satisfaction Variable (Agree-Disagree Scale)

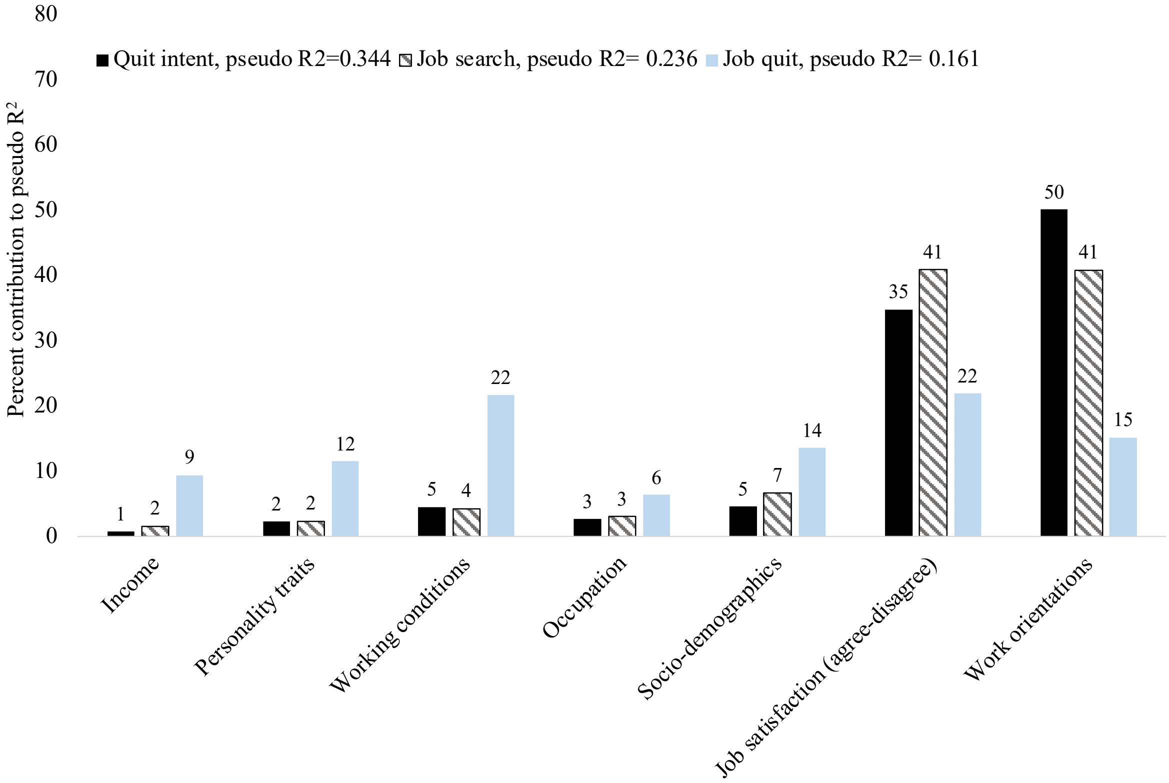


Notes: The Shapley decomposition quantifies each variable group's contribution to the overall model fit (pseudo$R^2$) while accounting for correlations among predictors. Values represent the percentage of explained variance attributable to each group. "Job characteristics" include working hours, public/private employee status, and tenure. The decomposition is based on Models (4)-(6) in Table C7. The difference with Figure 5 is the measure of job satisfaction; the figures are otherwise identical.

## APPENDIX D

## An archetypal typology of work orientations

We explore the existence of a typology of work orientations, with discrete types that reflect people's orientation towards work. Rather than considering the orientations as mutually exclusive and distinct dimensions, we adopt the so-called "archetypal analysis" that extracts profile-like types from our dataset (Cutler & Breiman, 1994; Eugster & Leisch, 2009). Cutler and Breiman (1994) and Eugster and Leisch (2009) offer a detailed discussion of the theoretical and statistical foundations of this statistical technique.

### Archetypal analysis

Archetype analysis is an explorative data reduction technique that identifies a few (not necessarily observed) observations located at the extreme ends of a multivariate dataset to represent all observations (Cutler & Breiman, 1994). Together, the few identified archetypes best approximate the dataset (i.e., the residual sum of squares is minimized) and are convex combinations of the points in the dataset (i.e., the archetypal coefficients are greater than or equal to 0 and sum to 1). The archetypal algorithm tries to satisfy these two principles and goes through several iterations to find the optimal solution. The optimal number of archetypes is typically selected by the decline in the RSS, up to the point that the decline is no longer statistically or practically significant (Beugelsdijk et al., 2022; Eugster & Leisch, 2009).

Once the optimal number of archetypes is determined, all observations in the dataset receive an archetypal score between 0-1 that resembles the overlap with the archetype. We visualize this in Figure D1. In this example, the dataset has an *x* and *y* dimension. The algorithm identifies three corner solutions as optimal (marked with a red x) in Panel B. Each of the individual observations (marked with the green dots) receives a score for each archetype depending on its position in the data cloud. Panel C displays that respondent $x_1$ fully resembles archetype 3 and will thus get a score of 1 for archetype 3 and a score of 0 for the other two archetypes. In contrast, respondent $x_3$, being positioned in the middle of the cloud, will get an almost equal score for each of the three archetypes.

Figure D1: Visualization of archetypal analysis

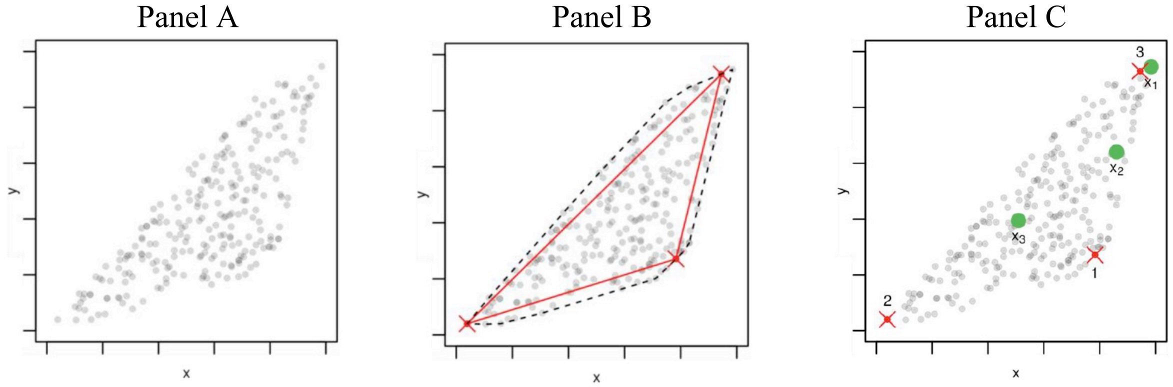


Unlike other data reduction techniques, such as factor analysis or cluster analysis, archetype analysis focuses on the observations rather than the distributional characteristics of the variables. The archetypal observations are selected in a way that retains as much of the information from the dataset. Once the archetypes are identified, each observation in the dataset is described by a weighted combination of the archetypes depending on their resemblance to the archetype. Observations are thus not classified into discrete groups, but are, by construction,

a *configuration* of archetypes. In contrast, data reduction techniques such as factor analysis estimate dimensions that explain a maximal degree of variance in the variables included in the analyses. Using these dimensions, each observation in the dataset receives a factor score that indicates the relative position of the observation vis-à-vis the average observation. Cluster analysis categorizes observations on a given set of characteristics (as determined by the researcher) into distinct groups that 'cluster' together, i.e., resemble each other.

**Measuring archetypes of work orientations**

We use the archetypes package in R to construct work orientation archetypes. Like in the main analyses, we use the 9 standardized work orientation items as input variables (Q4-Q6, Q8-Q13). To decide on the optimal number of archetypes, we inspect the scree plot and use the elbow criterion to determine the number of archetypes that does not further significantly decrease the RSS. Figure D2 shows a clear kink around three archetypes, with the first three archetypes contributing the most to the decrease in the RSS. We therefore select the model with three archetypes. This solution is not sensitive to the inclusion and exclusion of individual items or the use of different starting points of the algorithm.

Figure D2**:** Scree plot

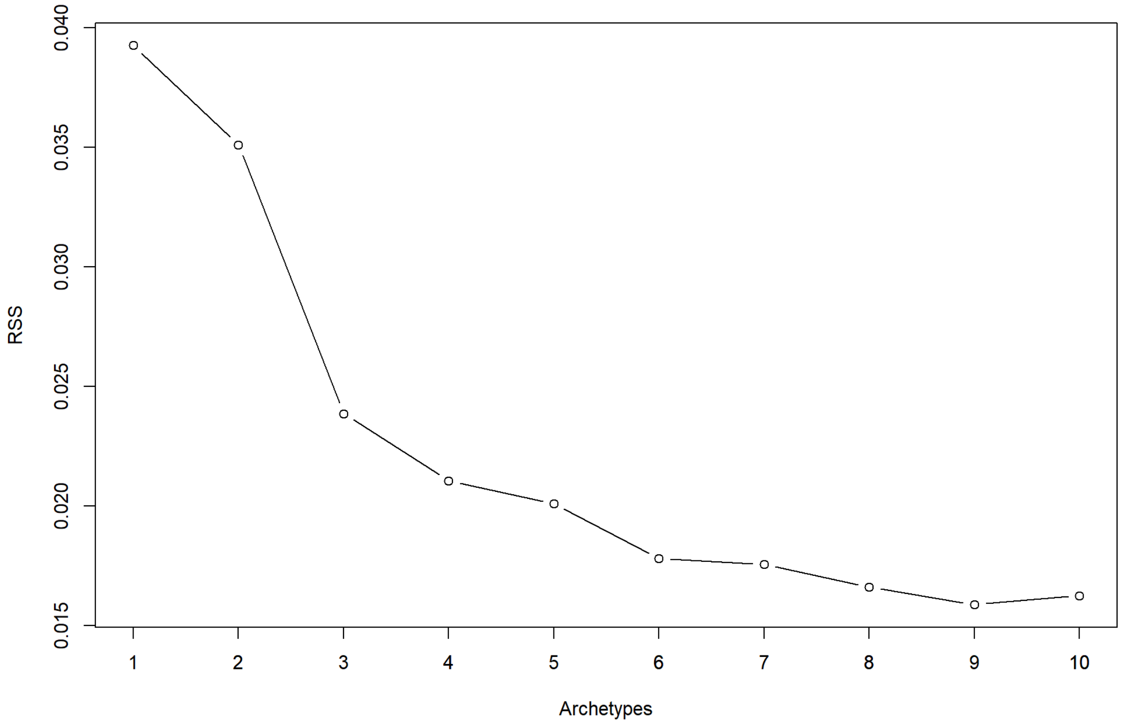


**Three archetypes of work orientations**

The three-archetype model consists of three archetypes that uniquely combine the 9 work orientation items. Based on the answers that each archetype gives to each of the 9 items, as well as the difference in answers across types, we can interpret each archetype. Table D1 displays the three archetypal configurations. *Archetype 1* indicates that their main reason for working is financial, to support their family and lifestyle (Q6). All other items are not applicable (at all). This is in line with the *job orientation. Archetype 2* attributes great importance to items Q11, Q12, and Q13, which are about the expectations to be in a higher-level job in five years, and about viewing the job as a stepping stone. Furthermore, respondents in this archetype do not expect to be in the same job in 5 years. This aligns with the *career orientation*. *Archetype 3* indicates that respondents enjoy talking about their work with others

(Q4), that their work is one of the most important things in their life (Q5), that they would continue in their job even if they would be financially independent (Q8), that their work makes the world a better place (Q9) and that they would choose their work again if they had the chance (Q10). This answer pattern aligns closely with having a *calling orientation*.

Table D1: Archetypal analysis of work orientation items: three archetypes

| | Archetype 1 (Job) | Archetype 2 (Career) | Archetype 3 (calling) |
|---|---|---|---|
| Q4 I enjoy talking about my work with others | -1.758 | -0.510 | 1.470 |
| Q5 My work is one of the most important things in my life | -1.707 | -0.855 | 1.900 |
| Q6 My main reason for working is financial: to support my family and lifestyle | 0.995 | 0.602 | -1.188 |
| Q8 If I was financially independent, I would continue my current work even if I wasn't getting paid for it | -1.124 | -1.020 | 1.938 |
| Q9 My work makes the world a better place | -1.728 | -0.365 | 1.575 |
| Q10 I would choose my current line of work again if I had the chance | -1.584 | -1.108 | 1.459 |
| Q11 I expect to be in a higher-level job in five years | -1.363 | 1.902 | -0.303 |
| Q12 I view my job as a stepping stone to other jobs | -1.143 | 2.277 | -0.695 |
| Q13 I expect to be doing the same work in five years (reversed) | -0.458 | 2.052 | -1.403 |

Note: N=1,748. All items are standardized with a mean of 0 and a standard deviation of 1.

Based on the answer pattern of all individuals in our sample, we measure the degree to which individuals resemble each archetype. This is reflected in archetypal scores, which by construction sum to 1. The distribution of types is shown in Figure D3. The average value of Archetype 1 is 0.30 (standard deviation 0.24), 0.29 for Archetype 2 (standard deviation 0.23), and 0.40 for Archetype 3 (standard deviation 0.21). This does not differ across the two analysis samples (see Figure D4).

The archetypal scores show a moderate to strong correlation with the three work orientation factors: AT1 correlates at 0.32 with job, AT2 correlates at 0.96 with career, and AT3 correlates at 0.92 with calling. Finally, examining the socio-demographic characteristics of each archetype reveals that women are more likely to have a calling orientation (AT3) than men. We see the opposite pattern for the career orientation (AT2). The career orientation (AT2) is more prevalent among younger respondents, whereas job (AT1) and calling (AT3) increase with age. Moreover, respondents with a college degree are more likely to have a career orientation (AT2), and respondents without such a degree are more likely to have a job orientation (AT1).

Figure D3: Ternary plot of the three work orientation archetypes

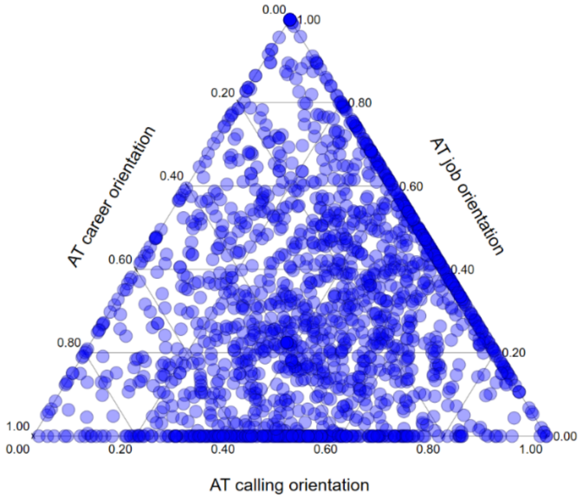


Notes: Each point in the figure represents an actual observation, i.e., a respondent, in our dataset. Their position is determined by their archetypal scores, which by construction sum to 1.

Figure D4: Distribution of work orientation archetypes across samples

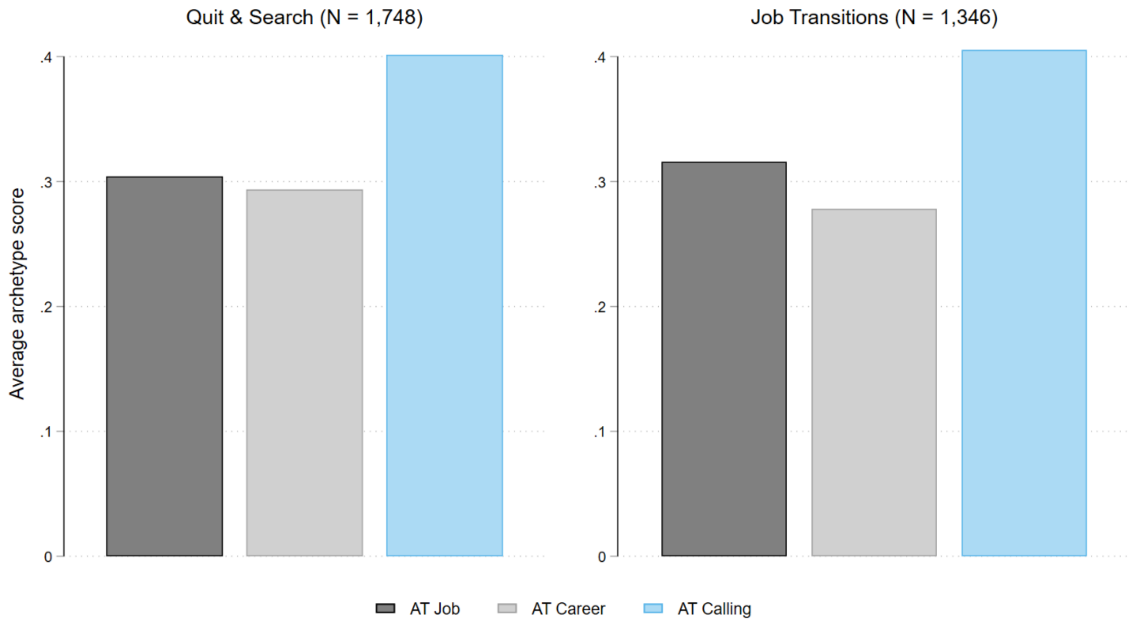


**Quit Intentions, Job Search, and Job Quitting**

After creating the archetypes and estimating the archetypal scores for each individual in our analysis dataset (N=1,748), we replicate the main analyses to see how the archetypes relate to quit intention, job search, and job quitting. The results are available in Table 7 in the main manuscript and summarized in Figure D5. They are in line with the main findings. Furthermore, Table D2 displays the Shapley decomposition results. We follow the approach from the main manuscript and use Shapley decompositions of the pseudo $R^2$. This decomposition is based on the fully controlled model (including controls for job satisfaction and personality traits). The results are in line with our main findings, confirming the relative importance of work orientations in explaining quit intent and job search behaviour.

Figure D5: The relationship between work orientations archetypes and quit intentions, job search, and job quits

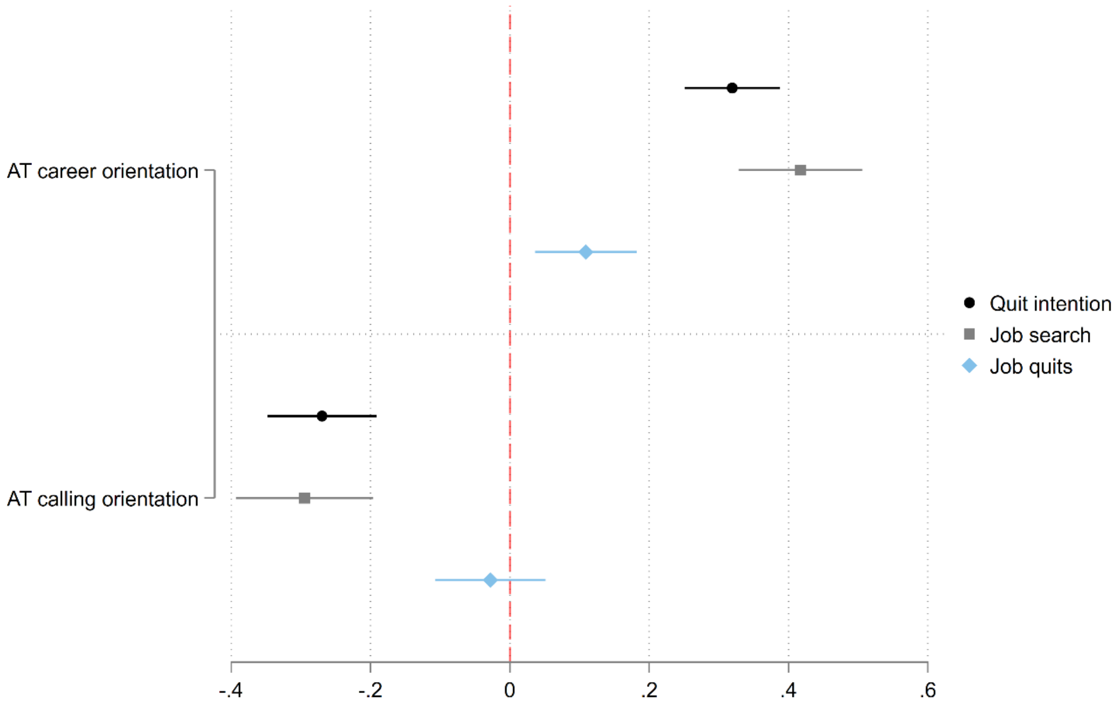


Notes: The plots are based on the coefficient estimates from Panel B, Columns (4)-(6) from Table 7. The coefficient estimates should be interpreted relative to the base category AT job orientation.

Table D2: Shapley decomposition

| | Quit intent | Job search | Job quits |
|---|---|---|---|
| Archetypes of work orientations | 46.95% | 40.93% | 14.08% |
| | | | |
| Income | 0.73% | 1.59% | 9.24% |
| Personality traits | 2.60% | 3.22% | 11.28% |
| Working conditions | 3.08% | 3.34% | 23.50% |
| Occupation | 3.40% | 3.40% | 11.27% |
| Socio-demographics | 3.95% | 6.05% | 11.03% |
| Job satisfaction (0-10) | 39.29% | 41.46% | 19.59% |

Notes: Quit intent, pseudo $R^2 = 0.339$; Job search, pseudo $R^2 = 0.223$; Job quit, pseudo $R^2 = 0.150$.

Continuing with Hypotheses 2-4, we interact each work orientation archetype with the relevant job characteristic to examine whether the effects of individuals' dominant work orientation on quitting behavior depend on job key characteristics (pay satisfaction, perceived career advancement, work meaningfulness). As a baseline model, we use the model from Panel A of Table 7 with the parsimonious set of controls (age and biological sex). Given that AT1 serves as the base category in this model, the coefficients on AT2 and AT3 reflect the effect of that archetype relative to the base category of AT1.

We first confirm that the work orientations, archetypes, and job characteristics are empirically distinct. The correlations between the job orientation archetype and pay satisfaction (r = –0.10) and between the career orientation archetype and perceived advancement opportunities (r = –0.05) are small. In contrast, the calling orientation archetype and work meaningfulness are strongly correlated (r = 0.65). This is in line with the main findings.

We report the interaction results in Table D3. The findings are largely in line with the findings in the main text, confirming that especially the career-oriented are more likely to (want to) leave or search for new jobs when promotion opportunities are limited. This is in line with H3. For job-oriented individuals, the interactions with pay satisfaction show little to no evidence of differential effects on quitting behavior. This also confirms the main results. Finally, we find that the calling orientation archetype is less likely to quit or leave their job at average work meaning. Yet this effect weakens when work is meaningful. This effect is strongest for quit intention and job search.

Table D3: Tests of Hypotheses 2-4 using the archetypes of work orientations

| | Quit intention (1) | Quit intention (2) | Job search (3) | Job search (4) | Job quit (5) | Job quit (6) |
|---|---|---|---|---|---|---|
| AT2 Career orientation | 0.454*** | 0.529*** | 0.534*** | 0.603*** | 0.138*** | 0.084* |
| | (0.043) | (0.052) | (0.051) | (0.062) | (0.042) | (0.044) |
| Poor advancement | -0.018** | -0.021** | -0.005 | -0.004 | -0.014 | -0.018** |
| | (0.008) | (0.009) | (0.012) | (0.012) | (0.009) | (0.009) |
| AT2 Career orientation × Poor advancement | 0.230*** | 0.249*** | 0.197*** | 0.199*** | 0.069** | 0.089*** |
| | (0.029) | (0.032) | (0.036) | (0.039) | (0.030) | (0.031) |
| AT3 Calling orientation | -0.092* | | -0.091 | | 0.048 | |
| | (0.051) | | (0.065) | | (0.048) | |
| Work meaningfulness | -0.060*** | -0.022** | -0.061*** | -0.022* | -0.030* | -0.007 |
| | (0.018) | (0.011) | (0.020) | (0.013) | (0.018) | (0.011) |
| AT3 Calling orientation × Work meaningfulness | 0.100*** | | 0.108*** | | 0.059* | |
| | (0.029) | | (0.035) | | (0.031) | |
| AT1 Job orientation | | 0.093* | | 0.096 | | -0.046 |
| | | (0.050) | | (0.065) | | (0.047) |
| Pay satisfaction | -0.008 | 0.005 | -0.022** | -0.028* | -0.021** | -0.000 |
| | (0.009) | (0.013) | (0.011) | (0.017) | (0.010) | (0.016) |
| AT1 Job orientation × Pay satisfaction | | -0.042 | | 0.013 | | -0.058 |
| | | (0.030) | | (0.035) | | (0.037) |
| Age | 0.000 | -0.000 | 0.000 | 0.000 | -0.002** | -0.002*** |
| | (0.001) | (0.001) | (0.001) | (0.001) | (0.001) | (0.001) |
| Male | -0.029** | -0.028** | -0.033* | -0.034* | -0.022 | -0.021 |
| | (0.014) | (0.014) | (0.018) | (0.018) | (0.014) | (0.015) |
| Constant | 0.005 | -0.059 | 0.060 | -0.000 | 0.102** | 0.165*** |
| | (0.050) | (0.046) | (0.065) | (0.059) | (0.050) | (0.046) |
| Observations | 1,537 | 1,537 | 1,537 | 1,537 | 1,266 | 1,266 |
| R-squared | 0.233 | 0.228 | 0.176 | 0.172 | 0.043 | 0.044 |

Notes: The reported estimates are obtained from OLS regressions including age and gender as controls. Pay satisfaction, perceived advancement opportunities, work meaningfulness are standardized to have a mean of 0 and a standard deviation of 1. The job orientation is omitted and served as based category in columns (1) (3) (5), and the calling orientation archetype is omitted and serves as the base category in columns (2) (4) (6). Robust standard errors in parentheses. * p<0.1, ** p<0.05, *** p<0.01.

**References for Appendix D**

Beugelsdijk, Sjoerd, Van Herk, Hester, & Maseland, Robbert (2022). The nature of societal conflict in Europe: an archetypal analysis of the postmodern cosmopolitan, rural traditionalist and urban precariat. *Journal of Common Market Studies,* 60(6), 1701-1722.

Cutler, Adele, & Breiman, Leo. (1994). Archetypal analysis. *Technometrics, 36*(4), 338-347.

Eugster, Manuel JA, & Leisch, Friedrich. (2009). From spider-man to hero—archetypal analysis in R. *Journal of Statistical Software, 30*, 1-23.
